# Men’s internet sex addiction predicts sexual objectification of women even after taking pornography consumption frequency into account

**DOI:** 10.3389/fpsyg.2025.1517317

**Published:** 2025-02-12

**Authors:** Pavla Novakova, Edita Chvojka, Anna Ševčíková, Lukas Blinka, Paul Wright, Steven Kane

**Affiliations:** ^1^Faculty of Social Studies, Masaryk University, Brno, Czechia; ^2^Department of Methodology and Statistics, Faculty of Social and Behavioural Sciences, Utrecht University, Utrecht, Netherlands; ^3^The University Graduate School, Indiana University Bloomington, Bloomington, IN, United States; ^4^Justice and Society, University of South Australia, Adelaide, SA, Australia

**Keywords:** internet pornography, internet sex addiction, sexual objectification, addiction, frequency

## Abstract

**Introduction:**

Problematic online video pornography consumption is associated with sexual objectification, particularly in male consumers. However, previous studies have not considered that there is a subgroup of internet users whose consumption may become problematic due to their internet sex addiction. Such users may, in response to internet sex addiction symptoms such as craving, have increased levels of sexual objectification.

**Methods:**

In a sample of 1,272 male consumers of online video pornography (Mage = 32.93, SDage = 9.44), we examined whether internet sex addiction is linked to sexual objectification via an online survey.

**Results:**

We fitted a series of structural equation models and found that men who scored higher on internet sex addiction were more likely to objectify women. More importantly, this link did not cease when controlling for the frequency of online video pornography consumption.

**Discussion:**

Our findings suggest that there are other mechanisms related to addictive symptomatology than just the link through online video pornography consumption that may contribute to sexual objectification. Addiction-related factors may have a unique role in fostering sexual objectification. Isolating internet sex addiction as a potential driver highlights the need to address objectifying behaviours in individuals struggling with this addiction.

## 1 Introduction

The sexually motivated use of the internet serves nowadays not only to seek sexual pleasure but also to avoid boredom or reduce stress (Böthe et al., 2021). Although people may search for information about sex, read erotic stories, have sex over a webcam or chat, search for sexual partners, and browse online sex shops (Ballester-Arnal et al., 2021), online video pornography (further just “pornography”) consumption remains currently the most prevalent activity (Wright et al., 2022; Hoagland and Grubbs, 2021), especially among male internet users.

Outcomes of frequent exposure to nudity and sexual activities (Campbell and Kohut, 2017) have been subject to a significant body of research (see Peter and Valkenburg, 2016; Harkness et al., 2015). Studies on the perceived positive effects of pornography consumption involve positive links with greater openness towards sexual experimentation, improved communication about sex, heightened sexual comfort, the exploration of sexual interests, understanding one’s sexuality, and better knowledge of anatomy, physiology, and sexual behaviour (Kohut et al., 2017; Hesse and Pedersen, 2017; Castro-Calvo et al., 2018). However, pornography consumption has also been researched in connection to adverse consequences, ranging from developing unhealthy sexual behaviour, such as internet sex addiction, to acquiring problematic sexual beliefs and attitudes toward the opposite gender (Baltazar et al., 2010; Harper and Hodgins, 2016; Herbenick et al., 2020; Lim et al., 2016; Paslakis et al., 2022; Wright et al., 2022).

Substantial research has shown the linkage between frequent pornography exposure and sexual objectification, particularly the treatment of women as sex objects (Wright and Tokunaga, 2016). While objectified individuals in pornography are not limited to women but also include men (Bridges et al., 2024) and people beyond the gender binary (Pavanello Decaro et al., 2024), we expand the research on objectification of women for several reasons grounded in the current literature. Research has consistently shown that pornography typically emphasises male pleasure, with a typical script focusing on male orgasms and close-ups of female bodies (Klaassen and Peter, 2015), which further reinforces the objectification of women. Furthermore, men who consume online pornography more frequently are more likely to perceive women as sex objects (Wright and Tokunaga, 2016; Bridges et al., 2024; Vandenbosch and van Oosten, 2017). This relationship is especially pronounced for male consumers, likely because pornographic content is often tailored toward male preferences (Miller and McBain, 2022) and because men tend to watch these videos more often and more intensely than women (Solano et al., 2020; Campbell and Kohut, 2017).

Frequent pornography use is not only typical behaviour for non-problematic pornography users but also for individuals at risk of internet sex addiction (Bőthe et al., 2020). Despite the growing body of research on sexual objectification, no studies to date have explored whether the sexual objectification exhibited by sex-addicted internet users exclusively relates to frequent pornography use or if it broadly connects to the symptoms of internet sex addiction. This pioneering research aims to examine whether internet sex addiction uniquely contributes to sexual objectification, even when accounting for the frequency of pornography use. By examining the issue of sexual objectification, we aim to foster a deeper understanding of the negative consequences experienced by individuals struggling with symptoms of internet sexual addiction.

Sexual objectification puts the physical appearance or sexual functionality of the body above the overall value of the person (e.g., Fredrickson and Roberts, 1997; Szymanski et al., 2011). An essential dimension of sexual objectification is the treatment of a person as a body (or its parts) whose only purpose is to provide sexual pleasure (Bartky, 2015). Although both men, women, and beyond binary-gendered people can be targets of sexual objectification, the concept is strongly associated with women (Rodríguez-Castro et al., 2018). Compared to men, images of women are more likely to display heightened attention to the body parts than the whole body (Gervais et al., 2012).

Many channels objectify women (e.g., TV programs, video games, and online media). However, one with the most potent effect is pornography (Willis et al., 2022). Klaassen and Peter (2015) concluded that women in pornographic material are more likely to be depicted in a reductive sense. A higher frequency of pornography consumption relates to a higher level of sexual objectification. Wright and Tokunaga (2016) found that the frequency of men’s exposure to online video pornography significantly correlated with objectified cognitions about women (which also predicted stronger attitudes supportive of violence against women). Vandenbosch and Eggermont (2019) revealed that adolescents are more likely to believe that women are sex objects if they consume sexually explicit internet materials more often. Frequent pornography consumption may, therefore, convey the acceptability of treating people, namely women, as sexual objects.

Some authors claim that frequent pornography use is also a common behaviour for people at risk of internet sex addiction (Bőthe et al., 2020). Problematic use of online materials has been conceptualised as a phenomenon that may fall within the hypersexual, compulsive-impulsive, or addictive spectrums of disorders (Fernandez and Griffiths, 2021). However, only Compulsive Sexual Behaviour Disorder, which includes problematic pornography use, has been determined to be an official diagnosis (World Health Organization, 2004), with problematic pornography use being its most important subset (Fuss et al., 2019; Sassover and Weinstein, 2022). Nevertheless, an increasing body of research suggests that problematic pornography use may indicate a behavioural addiction (Gola et al., 2017; Karila et al., 2014; Blinka et al., 2022). Authors supporting this thesis (Cooper et al., 2000; Schwartz and Southern, 2013; Orzack and Ross, 2000) argue that with the accessibility of pornography and other online sexual content, some internet users fail to control their pleasure-seeking or mood modification via pornography consumption to the extent that they fulfil other behavioural addiction criteria such as tolerance, withdrawal symptoms, and craving, ultimately leading to significant life problems (Griffiths, 2012; Cervero et al., 2024). Until now, the problematic pornography use was, according to the Compulsive Model, considered a tool to decrease anxiety, implying that some criteria and symptoms, e.g., pleasure-seeking, craving, or withdrawal, do not fit into this model. However, some studies (Blinka et al., 2022; Chen et al., 2018) showed that pleasure may be a strong trigger that drives internet users, including sex-addicted people, to search for sexual stimuli (e.g., to deliberately expose themselves to sexual stimuli to aid in achieving orgasm).

Incorporating a variety of behavioural theories, and information-processing theories (e.g., Bandura, 2014; Brown, 2002), the sexual script acquisition, activation, application model (3 AM) explains how and under what circumstances consuming pornography affects sexual cognition, including beliefs about sexual objectification (Wright and Tokunaga, 2016; Vandenbosch and van Oosten, 2017). Wright’s sexual script acquisition, activation, and application model (3 AM; Wright, 2011) integrates the sexual script theory (Simon and Gagnon, 2003; Huesmann, 1986) reasoning about mass media as a cognitive script provider. The 3 AM implies that pornography may modify consumers’ sexual cognition, including beliefs about sexual objectification, through the following mechanisms. Pornography users become exposed to scripts they are not aware of (acquisition) and primed with scripts of which they are already aware (activation). Moreover, pornography encourages the utilisation of such scripts by portraying specific sexual behaviours or patterns of sexual behaviour as rewarding, appropriate, and normative (application). Specifically, frequent exposure is thus likely to enhance long-term memory activation and make sex-objectifying scripts more accessible in situations when frequent pornography users evaluate their perception of and attitudes toward women (Wright, 2019).

There may be additional mechanisms at play in the heightened accessibility of objectified cognitions among addicted people. Specifically, in addicted people, objectification can also be maintained and strengthened as a response to cravings and experiences related to intentional or unintentional withdrawal from internet use and access to sexually explicit stimuli. Recent studies suggest that viewing women as sexual objects helps sexually addicted people cope with their irritation and craving from their lack of access to online pornography (Blinka et al., 2022; Ševčíková et al., 2018; Grubbs et al., 2019). In Blinka et al., participants acknowledged their tendency to *‘exploit sexual objects visually’* (i.e., to create a mental image full of sexual stimuli) when they did not have access to online pornography. The participants confided that, when abstaining, they were irritated by the lack of access to pornographic images to the extent that they tried to draw as much as possible from each potential sexual object (either in their environment or activated in their memory) to nourish their fantasy.

In this respect, sexually addicted men may adopt specific behavioural actions, such as walking near a nudist beach or leering at women from a balcony, to saturate their mental space with sexual imagery and relieve or avoid withdrawal symptoms (Ševčíková et al., 2018). Such maladaptive coping mechanisms may be particularly potent, as sexually addicted individuals demonstrate heightened neural responses to external erotic cues (Antons et al., 2020; Brand et al., 2016; Gola et al., 2017; Klein et al., 2022; Seok and Sohn, 2015). According to the incentive-sensitisation theory, repeated exposure to rewarding stimuli, such as pornography, results in neural alterations that make the individual hypersensitive to cues associated with sexual arousal and pleasure (Klein et al., 2022). This heightened sensitivity amplifies the salience of sexual stimuli, eliciting stronger reactions to sexual content (Love et al., 2015). As a result, individuals may disproportionately focus on and objectify physical attributes to achieve arousal (Robinson and Berridge, 2008). Due to this increased sensitivity to cues, sexually addicted people may visually “exploit sexual objects” (i.e., tend to objectify passers-by sexually) and construct vivid mental images rich in sexual stimuli during periods of abstinence.

In this respect, we propose to test the following hypotheses:

*H1*: Internet sex addiction significantly predicts sexual objectification with a small/medium positive effect.

*H2*: The relationship between internet sex addiction and sexual objectification remains significant even after controlling for online video pornography consumption frequency.

Should the relationship between internet sex addiction and sexual objectification remain significant even after controlling for the frequency of online pornography consumption, we would conclude that there may be additional mechanisms beyond frequent pornography exposure in the relationship between sex addiction and sexual objectification. Since men generally consume pornography more frequently and intensely than women (Campbell and Kohut, 2017; Solano et al., 2020), with content predominantly tailored to male preferences, emphasising male satisfaction and the sexual objectification of women (Klaassen and Peter, 2015; Miller and McBain, 2022; Rodríguez-Castro et al., 2018; Gervais et al., 2012), this study focuses on self-identified heterosexual male porn consumers, who may be particularly susceptible to sexual objectification.

## 2 Materials and methods

### 2.1 Data collection and participants

The data come from a convenience sample online survey about internet sex conducted in the Czech Republic in 2017. The study was primarily advertised on the biggest Czech online erotic platform, Amateri.com (2,215 participants, which is 88% of the whole sample, came from this website) and via social networking sites that targeted respondents who engaged in online sexual activities. The Czech Republic is racially and ethnically homogeneous and, like other European countries, has a large percentage of internet users (ČSÚ, 2024). The Czech Republic is also known to be one of the most secular countries in Europe with less conservative views towards family, marriage, and sexual norms (Chromková Manea and Rabušic, 2021).

A total of 2,518 participants aged 18 to 77 (Mage = 32.73, SDage = 9.62; 73.2% men) filled in the questionnaire in the Czech language, indicating that the users of Amateri.com are predominantly young heterosexual men. There were 1,312 non-homosexual men in total; no man indicated bisexuality. For this study, we worked with a subset of 1,272 men (Mage = 32.93, SDage = 9.44) who self-identified as heterosexual, who filled in more than 75% of the questionnaire, and whose age distribution aligned with the whole sample. The choice of 75% reflected our motivation to avoid the inclusion of careless, bored, or otherwise demotivated participants and related response biases. We chose the 75% completion threshold to balance data completeness with retaining as much sample as possible for robust statistical analysis. Moreover, we wanted to ensure the validity of the multiple imputation, which was part of our data preparation routine, and which replaces the missing values with those predicted from the remaining data entries. The 75% level allowed the inclusion of participants who meaningfully engaged with all constructs.

The data collection occurred from April to November 2017 on the Lime Survey platform. The participants were informed about the purpose of the study, data management and analytical procedures, and their rights. This study was conducted per the university’s ethical guidelines (Ethics Committee statement can be found in the Attachments). Details about the study aim, procedures, data collected, and the minimum age (18 years old) were provided on the first page of the questionnaire. Participation was voluntary and all information provided was confidential. We did not collect any personal data, and no incentives were offered to participants. A written informed consent was also obtained. The survey was conducted as part of a research project funded by the Czech Science Foundation.^1^

### 2.2 Measures

#### 2.2.1 Internet sex addiction

Internet sex addiction in the preceding 12 months was measured with the Short French Internet Addiction Test Adapted to Online Sexual Activities (s-IAT-sex; Wéry et al., 2016). The scale assesses the potential symptoms of addiction to sexual websites. Wéry et al. developed the instrument to adapt the general IAT (Pawlikowski et al., 2013). The Czech version used in this survey underwent a 3-way back-translation process. The tool consists of 12 items with a 5-point Likert scale that ranges from “never” (Böthe et al., 2021) through “rarely,” “sometimes,” “often” to “very often” (Campbell and Kohut, 2017). The items tap into the core criteria of addiction. The scale has two dimensions: (1) the loss of control and time management: e.g., “How often do you find that you stay on internet sex sites longer than you intended?” and (2) craving and social problems: e.g., “How often do you choose to spend more time on internet sex sites than going out with others?” measured by six items each. According to Wéry et al., the tool shows good psychometric properties. However, the correlation between the two latent factors in the original study was extremely high (0.89), and recent literature, including the authors themselves (e.g., Chen and Jiang, 2020; Levi et al., 2020; Wéry et al., 2018; Wéry et al., 2020), has exclusively treated s-IAT-sex as unidimensional. This opens the question of whether the two factors represent distinct dimensions. We also decided to treat the scale as unidimensional (which deviated from our pre-registration of the original two-dimensional model), as alternative two-dimensional models either fitted the data poorly overall or exhibited non-significant factor loadings in the loss of control and time management dimension, which also suggests the lack of overlap between the model and the data (see Results and Discussion for more elaboration). The reliability of the general factor was high (McDonald’s ω_total_ of 0.89). The distributions of most s-IAT-sex items were notably skewed (skewness: mean = 1.33, median = 1.37, min = −0.04, max = 2.52; kurtosis: mean = 1.61, median = 1.29, min = −0.77, max = 6, 39). This indicates that s-IAT-sex would prevent the pathologising of internet use for sexual purposes but, consequently, was not able to discriminate between the different levels of addiction severity.

#### 2.2.2 Sexual objectification

Sexual objectification was measured by five items (full version in Appendix) that addressed the frequency of typical objectifying behaviour (e.g., scanning someone’s body, undressing passersby with eyes, observing a body instead of listening, having lustful glances, and observing certain parts of others’ bodies). The respondents indicated the frequency of such behaviour with a 5-point Likert scale, going from “never” through “rarely,” “sometimes,” “often” to “very often.” The item wording was based on semi-structured interviews with sexually addicted men who searched for treatment (Ševčíková et al., 2018). The responses were fairly normally distributed (skewness: mean = −0.09, median = 0.09, min = −0.68, max = 0.23; kurtosis: mean = −0.49, median = −0.52, min = − 0.83, max = 0.12). We treated sexual objectification as a latent trait for our analysis and estimated a one-factor model over the five items. The measure’s reliability was high (McDonald’s ωtotal of 0.86).

#### 2.2.3 Frequency of online video pornography consumption

The online porn consumption was captured by one item: “How often have you used the internet to watch online video pornography in the last 12 months?” The participants answered on a 7-point Likert scale going from “daily” (=1) through “two times to five times a week,” “once a week,” “two times or three times a month,” “once a month,” “less than once a month” to “not once” (=7), and the item was re-coded so that (Böthe et al., 2021) would indicate “not once” and (Harkness et al., 2015) would indicate “daily.” The responses were fairly skewed (skewness = −1.52, kurtosis = 2.52).

### 2.3 Data cleaning and preparation

We started with the general sample. We excluded all women and homosexual men. We then excluded people with more than 25% missing responses. Subsequently, we imputed the missing data points with the CART method (Van Buuren and Groothuis-Oudshoorn, 2011), which is a procedure with an origin in machine learning that is based on classification trees that perform well with heavily skewed data (Burgette and Reiter, 2010), such as the responses on the s-IAT-sex items. Moreover, while our analysis technique could handle incomplete data, missing responses can still bias the estimates, which we wanted to prevent. We then performed a non-paranormal transformation to move the data distribution closer to normal (*gaussianization*, Zhao et al., 2011). We checked for outliers by visually inspecting boxplots and performing Grubb’s tests (Komsta, 2022) and found none. This left 1,272 men. We re-ran the pre-registered sensitivity analysis (Moshagen, 2021) because the resulting sample was smaller than expected. The analysis indicated a power of ~1 to detect a small effect.

### 2.4 Data analysis

The analysis was pre-registered via the Open Science Framework (OSF - unblinded).^2^ The analyst had not seen the data beyond the “variable view” in SPSS before downloading them once the registration was approved. The full dataset is not publicly available per the PI’s decision. However, a subset of the data used for this study is available in the associated OSF repository, with the analytic script and a basic codebook. All analyses were conducted in R version 4.2.1 (R Core Team, n.d.) and used the lavaan package (Rosseel, 2012).

We used Structural Equation Modelling to test our hypotheses. Since gaussianization was ineffective in transforming the heavily skewed variables, we used a robust weighted least square mean and variance-adjusted estimation method (WLSMV) and polychoric correlations as input. The reason to use an estimation method geared towards categorical data was based on two important characteristics of our data. First, both scales in our study utilised a 5-point Likert scale, which lies at the boundary that separates data that can be treated as continuous from categorical data (DiStefano and Morgan, 2014; Maydeu-Olivares et al., 2017; Rhemtulla et al., 2012). However, substantive skewness in some of our data (i.e., the s-IAT-sex responses) supported our decision to treat the data as categorical and avoid methods such as maximum likelihood, which assume data that are continuous and normally distributed. We identified all models by setting latent variances to one. We first established the measurement models by conducting CFAs. Then, we fitted Model 1 with only one direct regression path from Internet sexual addiction to Sexual Objectification. Then, we fitted Model 2 with an additional indirect path via the frequency of online video pornography consumption. Model 1 was nested in Model 2 as it included a subset of the parameters of Model 2. Thus, we conducted a χ^2^ difference test to assess whether adding the indirect path significantly improved fit.

### 2.5 Outcome measures

#### 2.5.1 χ^2^ difference test

We were interested in whether adding the indirect path via the frequency of online video pornography consumption would improve our model’s fit. If one structural equation model contains a subset of parameters of another (i.e., it is ‘nested’ in the model with more parameters), one can directly subtract the models’ χ^2^ values and degrees of freedom and statistically test for the significance of this difference. Model 1 (with only the direct path from Internet sexual addiction to Sexual Objectification) is nested in Model 2 (with both the direct regression from Internet Sex Addiction to Sexual Objectification, as well as the indirect effect via the frequency of consumption). We, therefore, subtracted the two χ^2^ statistics and the models’ degrees of freedom from each other and evaluated the significance of this difference. We flagged the model with the lower χ^2^ as better fitting in case of a significant difference, meaning that we prefer this model over the one with the higher χ^2^ value, as it explains significantly more variance in the data.

#### 2.5.2 Goodness-of-fit indices

Next to the χ^2^ statistic, we report RMSEA and its 95% CI, CFI, TLI, and SRMR. We pre-registered the following criteria to flag a good-fitting model: RMSEA <0.08, CFI and TLI > 0.9, and SRMR <0.1. Nevertheless, it is worth mentioning that the conventional goodness-of-fit guidelines, such as those by Hu and Bentler (1999), have been criticised for their lack of generalizability, as their values are sensitive to other model characteristics than the amount of misspecification (see McNeish, 2023; McNeish and Wolf, 2023; Fan and Sivo, 2007). We, therefore, interpret the fit indices only as a rough indication.

#### 2.5.3 Bootstrap confidence intervals

We also report the 95% bootstrap confidence around the regression coefficients over 1,000 resamples to assess the significance of the direct and indirect effects. Should the CIs contain zero, we deem the regression coefficient non-significant.

## 3 Results

### 3.1 Descriptive statistics

Descriptive statistics after multiple imputation and gaussianization can be found in Appendix A.

### 3.2 Measurement models

We first estimated a model that treated internet sex addiction as a second-order factor over the two dimensions (*loss of control/time management* and *craving/social problems*), as defined by Wéry et al. (2016). While this model fitted the data well, none of the loadings in the loss of control/time management dimension was significant (see Appendix B). Since discarding all the items with non-significant loadings would lead to omitting the original *loss of control/time management* dimension, we decided to deviate from the pre-registration and estimate an alternative model. We first fixed both paths from the second-order factor to the first-order factors to 1, estimating a model equivalent to a two-factor solution with correlated factors. This remedied the problem with the non-significant loadings. However, the resulting model did not fit the data (see Table 1 and Appendix C). Finally, we discarded the second-order factor and fitted a model with one general factor of internet sex addiction, which loaded on all items of s-IAT-sex. This model fitted the data satisfactorily (see Table 2) and showed substantial factor loadings (ranging from 0.57 to 0.83, mean = median loading = 0.69). We used the one-factor measurement model for testing our hypotheses. The one-factor model of Sexual Objectification fitted the data well (see Table 2) and showed substantial factor loadings (ranging from 0.61 to 0.76, mean = median loading = 0.70).

**Table 1:** Model fit and comparison.

	χ^2^	p(χ^2^)	df	RMSEA 90% CI lower	RMSEA	RMSEA 90% CI upper	CFI	TLI	SRMR
Hypothesis testing									
Model 1: only direct path	1159.32	<0.01	135	0.07	0.08	0.08	0.98	0.97	0.08
Model 2: direct + indirect path	558.09	<0.01	133	0.05	0.05	0.05	0.99	0.99	0.05
Difference	128.73	2	<0.01						
Measurement models									
s-IAT-sex 2nd order	503.01	<0.01	116	0.05	0.05	0.06	0.99	0.99	0.05
s-IAT-sex 2-factor	2515.88	<0.01	118	0.12	0.13	0.14	0.94	0.93	0.11
s-IAT-sex 1-factor	538.57	<0.01	118	0.05	0.05	0.06	0.99	0.99	0.05
objectification	36.82	<0.01	5	0.05	0.07	0.09	0.99	0.99	0.03

2nd order = second-order model with two first-order factors based on Wéry et al. (2016); 2-factor = model equivalent to a two-factor solution with correlated factors based on Wéry et al. (2016).

**Table 2:** Descriptive statistics before multiple imputation and transformation.

	Mean	sd	Median	Min	Max	Skewness	Kurtosis
Age	32.92	9.44	31	18	77	0.88	0.89
Frequency of online pornography consumption	5.84	1.22	6	1	7	−1.52	2.52
Internet sex addiction S-IAT-SEX							
C1 Covers visiting sex pages	1.81	1.11	1	1	5	1.28	0.74
C2 Angry when disturbed from sex pages	1.36	0.79	1	1	5	2.52	6.39
C3 Thinks of sex activities on internet	1.86	0.92	2	1	5	0.89	0.13
C4 Hides the amount of time spent on sex pages	1.70	1.12	1	1	5	1.59	1.46
C5 Does not go out because of sex pages	1.66	0.94	1	1	5	1.41	1.45
C6 Does not feel himself without sex pages	1.37	0.76	1	1	5	2.31	5.32
L1 Visits sex pages for longer than intended	2.80	1.08	3	1	5	−0.04	−0.77
L2 Neglects household	1.69	0.93	1	1	5	1.32	1.20
L3 Limited work performance	1.50	0.81	1	1	5	1.71	2.61
L4 Limited sleep because of sex pages	1.98	1.04	2	1	5	0.86	0.00
L5 Wants to stay “just a few more minutes”	2.12	1.15	2	1	5	0.69	−0.57
L6 Unsuccessful at limiting sex pages	1.59	0.89	1	1	5	1.46	1.38
Objectification							
O1 Scans someone’s figure with his eyes	3.93	0.91	4	1	5	−0.68	0.12
O2 Creepy gaze	2.77	1.13	3	1	5	0.11	−0.73
O3 Observes select body parts	3.30	1.02	3	1	5	−0.20	−0.49
O4 Observes body parts instead of listening	2.66	1.05	3	1	5	0.23	−0.52
O5 Undresses passers−byes with his eyes	2.86	1.18	3	1	5	0.09	−0.83

Items labelled “L” originally fall to the loss of control/time management factor, items labelled “C” originally fall into the craving/social problems factor of S-IAT-SEX (Wéry et al., 2016).

### 3.3 Hypothesis testing

**Figure 1:**
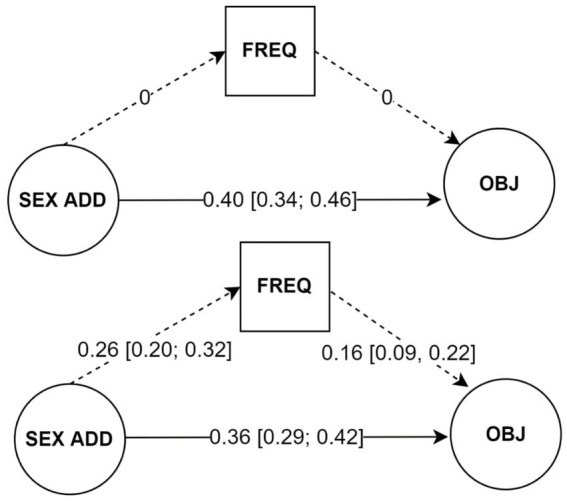
Fully standardised regression coefficients of Model 1 (upper panel) and Model 2 (lower panel). Only structural part of the SEM models is displayed. Latent (fixed to 1) and residual variances omitted for clarity. 95% bootstrap CIS (1,000 resamples) in squared brackets, zero within the bootstrap CI bounds would indicate a non-significant path. In Model 1 the indirect path via frequency (dotted lines) is closed. In Model 2 the indirect path via frequency is open. SEX ADD, internet sex addiction; FREQ, frequency of online pornography consumption; OBJ, sexual objectification.

The two models are compared in Table 1 and Figure 1. Model 2 (with the indirect path *internet sex addiction - > frequency - > sexual objectification* added on top of the direct path *internet sex addiction - > sexual objectification*) fitted the data significantly better than Model 1 (only the direct path *internet sex addiction - > sexual objectification*). Model 2 also fitted the data well in terms of the fit measures. Moreover, opening the indirect path via the frequency of consumption did not substantively weaken the direct (moderately strong) relationship between internet sex addiction and sexual objectification. Internet sex addiction predicted the frequency of consumption with a small effect, and the frequency of consumption predicted sexual objectification with a medium-sized effect. Thus, we supported both our hypotheses. A complete overview of all the estimated parameters is in Supplementary Appendix D.

## 4 Discussion

In this study, we investigated whether there may be additional mechanisms beyond the frequency of frequent pornography exposure in the relationship between sex addiction and men’s sexual objectification of women. In a sample of heterosexual men who used the internet for sexual purposes, we found a relationship between internet sex addiction and sexual objectification (SO). This direct relationship remained virtually unchanged even after opening the indirect path between online sex addiction and sexual objectification through the frequency of pornography consumption. Internet sex addiction was thus directly associated with sexual objectification, regardless of the frequency of consumption, suggesting that factors other than exposure to pornography are also at play in men’s objectification of women. Our finding of a relatively strong and significant association between internet sex addiction and sexual objectification with pornography use frequency simultaneously considered contributes to the current understanding by suggesting that sexual objectification is not solely a result of frequent pornography consumption. The finding is in line with prior research according to which sexually addicted men experienced intense urges to exploit sexual objects (i.e., women) visually when they did not either purposefully or unintentionally have access to pornography (Blinka et al., 2022). Ševčíková et al. (2018) suggest that, in this context, extensive sexual objectification may function as a withdrawal-related behaviour to cope with craving when pornography is avoided or inaccessible. This extensive sexual objectification allows sexually addicted people to saturate mental content with sexual stimuli to address sexual urges and achieve pleasure.

The direct link between internet sex addiction and sexual objectification may also point towards other mechanisms that connect internet sex addiction and sexual objectification and, therefore, explain the direct relationship. One such mechanism is the reward-seeking behaviour commonly associated with addiction. In line with the incentive-sensitisation theory (Robinson and Berridge, 2008), individuals with sexual addiction may develop an intensified response to sexual stimuli due to their brain’s heightened sensitivity to these cues. This theory posits that repeated exposure to rewarding stimuli, such as pornography, results in neural alterations that make the individual hypersensitive to cues associated with sexual arousal (Klein et al., 2022). Over time, these cues, even in the absence of pornography, can evoke strong cravings, leading to compulsive behaviours such as objectifying others to satisfy heightened sexual urges (Prause et al., 2015). This increased sensitivity makes sexual stimuli more salient, resulting in stronger reactions to sexual content (Love et al., 2015) causing individuals to disproportionately focus on and objectify physical attributes as a way to achieve arousal (Böthe et al., 2021). Consequently, sexual objectification can be viewed as a maladaptive reward-seeking behaviour driven by the brain’s sensitised reward system (Chau et al., 2024). Increased sensitivity to cues may lead to sexual objectification even in the absence of active pornography consumption.

It is also possible that a long-sustained and deeply internalised habit of sexual objectification due to frequent pornography use may make men susceptible to frequent thinking of any sexual activity and people as sexual objects (Willis et al., 2022). If pornographic material is not accessible, a natural reaction is to seek it elsewhere to facilitate masturbation, which has been recognised as a strategy to deal with withdrawal symptoms when access to pornography is restricted (Laier et al., 2013; Novakova et al., 2024).

However, beyond addiction mechanisms, it is crucial to consider cultural and gender norms as an alternative explanation for these findings. Sexual objectification may be shaped by broader socio-cultural frameworks that normalize and perpetuate objectifying attitudes and behaviours (Loughnan et al., 2011; Ward et al., 2023). For example, cultural and gender scripts that portray men as sexually dominant and women as passive objects of desire may reinforce objectification patterns over time (Seabrook et al., 2016). In addition, societal norms may make heterosexual men susceptible to frequent pornography use and consequently at risk of sex addiction and sexual objectification. These societal norms may amplify the association between internet sex addiction and sexual objectification. Understanding how cultural contexts interact with addiction mechanisms could provide a more comprehensive explanation for the observed link.

Apart from the main findings, this study has two additional methodological outcomes. First, the 5-item scale for sexual objectification developed for the present study showed desirable properties in terms of high and significant factor loadings and high internal consistency. After more thorough construct validation, the short scale can be adopted by other researchers and aid future research on sexual objectification. Second, our analyses did not support the two dimensions of s-IAT-sex as proposed by Wéry et al. (2016). This finding aligns with other studies (e.g., Chen and Jiang, 2020; Levi et al., 2020; Wéry et al., 2018; Wéry et al., 2020). The original work of Wéry et al. reported a substantial correlation between the two dimensions, leading to whether it is plausible to expect the s-IAT-sex instrument to fit a two-dimensional model. In this respect, we do not attribute the unsatisfactory behaviour of two-dimensional models to possible cultural differences but rather to the model itself. Treating the measure (and the construct) as two-dimensional could have severe ramifications if repeatedly demonstrated problematic across multiple studies. The difficulties with the two-dimensional model may suggest further refinement, potentially integrating the cognitive-behavioural model of pathological use (Bőthe et al., 2020), which separates cognitive preoccupation from behavioural consequences. This model may offer additional insights into how sex addiction manifests concerning objectification tendencies. For now, we encourage other researchers to revisit the theory behind s-IAT-sex and advocate for treating s-IAT-sex as unidimensional, as suggested in the study by Chen and Jiang (2020), until an alternative dimensional structure is proposed.

Our study is not without limitations. Since we only collected cross-sectional data, the causal pattern behind the observed link must be investigated in future studies. Sexual objectification in the form of internalised gendered norms may be a precursor for internet sex addiction. The two constructs can also reciprocally reinforce each other, creating a feedback loop. While the inclusion of the indirect relationship through the frequency of pornography consumption is an important first step, it does not allow for any temporal or causal conclusions. Therefore, incorporating longitudinal or experimental designs in future studies to establish how the relationship unfolds over time is of paramount interest. A cross-lagged panel design may explore the relationships between internet sex addiction, sexual objectification and frequency of porn consumption in a fashion similar to our study, yet over several measurement waves. Such a setting would allow researchers to examine whether addiction symptomatology predicts sexual objectification on the following measurement occasion or vice versa, as well as examine the role of porn consumption frequency in these temporal transitions. Furthermore, the growing popularity of the experience sampling methodology (ESM) may allow researchers to examine the relationship on an even more granular scale by logging objectification, addiction symptomatology and frequency of porn consumption in a diary-like fashion. Also, (quasi)experimental designs targeted at interventions limiting pornography consumption that would capture objectifying behaviours and addiction symptomatology may also provide insights into the temporal order of the relationship. Furthermore, it is important to note that both the s-IAT measure and the measure of pornography use frequency tap exposure to pornography. Thus, while including the pornography use frequency measure as a covariate furthers the goal of assessing the offline effects of internet sex addiction on men’s sexual objectification of women, it would be an inferential error to conclude that this association is wholly disconnected from the effects of men’s pornography viewing on their objectified cognitions.

The sexual objectification measure constructed for this study was undirected concerning different forms of sexual objectification, as well as the gender identity of the objectified. Different forms of sexual objectification, ranging from visual to verbal to behavioural objectification, may operate in various ways depending on the level of addiction severity. Future studies could examine whether specific types of objectification are more strongly linked to addiction symptomatology, adding granularity to the understanding of how addiction and objectification interact.

Moreover, the findings of this study are specific to heterosexual male consumers of pornography. Future research is needed to explore whether the relationship between sex addiction and sexual objectification persists among women and non-heterosexual pornography users. In addition, men who self-identify as heterosexual do not exclusively engage with heterosexual pornography (Sharkey et al., 2022) and may also objectify people of other genders (Engeln-Maddox et al., 2011). Since our measure did not capture these nuances, the relationships may be biased when only women are the target of sexual objectification. We worked with a robust, convenient sample of internet users who visited sexually oriented websites but experienced little to no symptoms of internet sex addiction. Those with intense addiction symptomatology may refrain from visiting pornographic sites to self-cure or may be less open about their problematic sexuality. Thus, future studies may find differences in clinical samples. Additionally, the five-item gender-neutral objectification measure, developed based on interviews with sexually addicted men (Ševčíková et al., 2018), has yet to be validated. However, this instrument demonstrated favourable properties within the structural equation models (SEM), including high and significant factor loadings and strong reliability. Although we also used a single-item measure of online pornography consumption, this is a typical approach for studying sexual behaviour in population-based surveys (e.g., Smith et al., 2003). Lastly, although the data were collected in 2017, the study addresses phenomena such as internet sex addiction, pornography use, and sexual objectification, which remain pressing issues even today.

In conclusion, this research highlights that addiction-related symptomatology may have a unique role in fostering sexual objectification. Isolating internet sex addiction as a potential driver may expand the scope of inquiry in the field of sexual behaviour and objectification and highlight the need to address objectifying behaviours in individuals struggling with internet sex addiction to improve their treatment outcomes. Specifically, treatment approaches should focus on addressing distorted beliefs and their role in the behavioural actions associated with abstaining from online pornography while also equipping clients with healthier coping skills (Wilson, 2010). Our findings indicate a need for future studies to explore how addiction-related mechanisms, such as cognitive distortions and cue sensitivity, may contribute to sexual objectification tendencies directed at women and also men and individuals beyond the gender binary. Ultimately, such findings could have vast implications for clinical interventions and broader societal discussions around media consumption and gender relations among diverse internet users. These findings are highly relevant to society as they reveal how internet sex addiction can drive sexual objectification, reinforcing harmful gender stereotypes and contributing to broader issues of inequality (Loughnan et al., 2011). They suggest practical ways to improve prevention and treatment strategies for internet sex addiction and sexual objectification. For instance, media literacy programs help people to be more aware of their perceptions of gender stereotypes (Walsh et al., 2014). Similarly, Pinkleton et al. (2012) found that media literacy training for teenagers reduced their acceptance of sex myths. Also, other researchers (Austin et al., 2015) found that such lessons weakened the effect of unrealistic media messages about sex in their decision-making process. In conclusion, thanks to media literacy programs, people recognise and challenge the objectifying messages commonly found in pornography, fostering healthier attitudes toward gender and sexuality (Vandenbosch and van Oosten, 2017).

## Data Availability

The original contributions presented in the study are included in the article/Supplementary material, further inquiries can be directed to the corresponding author.

## Footnotes

^1^ https://starfos.tacr.cz/en/projekty/GA15-19221S?query=2piqaac72svq

^2^ https://osf.io/n4rvb
