# Supplementary material for: Men’s internet sex addiction predicts sexual objectification of women even after taking pornography consumption frequency into account
Source: Front Psychol. 2025 Feb 12;16:1517317. doi: 10.3389/fpsyg.2025.1517317 (PMC11861099; doi:10.3389/fpsyg.2025.1517317)
Supplement: Supplementary file 2 [file Data_Sheet_2.pdf]

Appendix 1a: Descriptive statistics before multiple imputation and gaussianization

vars	n	mean	sd	median	trimmed	mad	min	max	range	skew	kurtosis	se
id	1272	4620,03	2485,79	4371,00	4628,81	3194,26	53,00	8915,00	8862,00	0,03	-1,19	69,70
sex	1272	1,00	0,00	1,00	1,00	0,00	1,00	1,00	0,00			0,00
age	1272	32,92	9,44	31,00	32,09	8,90	18,00	77,00	59,00	0,88	0,89	0,26
orientation	1272	1,00	0,00	1,00	1,00	0,00	1,00	1,00	0,00			0,00
freq	1270	5,84	1,22	6,00	6,05	1,48	1,00	7,00	6,00	-1,52	2,52	0,03
s_iat_sex1_loss	1269	2,80	1,08	3,00	2,82	1,48	1,00	5,00	4,00	-0,04	-0,77	0,03
s_iat_sex2_loss	1272	1,69	0,93	1,00	1,53	0,00	1,00	5,00	4,00	1,32	1,20	0,03
s_iat_sex3_loss	1271	1,50	0,81	1,00	1,32	0,00	1,00	5,00	4,00	1,71	2,61	0,02
s_iat_sex4_cra	1268	1,81	1,11	1,00	1,59	0,00	1,00	5,00	4,00	1,28	0,74	0,03
s_iat_sex5_cra	1271	1,36	0,79	1,00	1,16	0,00	1,00	5,00	4,00	2,52	6,39	0,02
s_iat_sex6_loss	1267	1,98	1,04	2,00	1,83	1,48	1,00	5,00	4,00	0,86	0,00	0,03
s_iat_sex7_cra	1270	1,86	0,92	2,00	1,74	1,48	1,00	5,00	4,00	0,89	0,13	0,03
s_iat_sex8_loss	1271	2,12	1,15	2,00	1,99	1,48	1,00	5,00	4,00	0,69	-0,57	0,03
s_iat_sex9_loss	1270	1,59	0,89	1,00	1,42	0,00	1,00	5,00	4,00	1,46	1,38	0,03
s_iat_sex10_cra	1267	1,70	1,12	1,00	1,45	0,00	1,00	5,00	4,00	1,59	1,46	0,03
s_iat_sex11_cra	1265	1,66	0,94	1,00	1,50	0,00	1,00	5,00	4,00	1,41	1,45	0,03
s_iat_sex12_cra	1266	1,37	0,76	1,00	1,18	0,00	1,00	5,00	4,00	2,31	5,32	0,02
with_1	1260	1,60	0,86	1,00	1,44	0,00	1,00	5,00	4,00	1,38	1,32	0,02
with_2	1265	1,34	0,71	1,00	1,16	0,00	1,00	5,00	4,00	2,14	3,95	0,02
with_3	1265	1,56	0,86	1,00	1,40	0,00	1,00	5,00	4,00	1,48	1,50	0,02
obj_1	1180	3,93	0,91	4,00	4,01	1,48	1,00	5,00	4,00	-0,68	0,12	0,03
obj_2	1179	2,77	1,13	3,00	2,76	1,48	1,00	5,00	4,00	0,11	-0,73	0,03
obj_3	1171	3,30	1,02	3,00	3,30	1,48	1,00	5,00	4,00	-0,20	-0,49	0,03
obj_4	1179	2,66	1,05	3,00	2,65	1,48	1,00	5,00	4,00	0,23	-0,52	0,03
obj_5	1179	2,86	1,18	3,00	2,83	1,48	1,00	5,00	4,00	0,09	-0,83	0,03

Appendix 1b: Descriptive statistics after multiple imputation and gaussianization

vars	n	mean	sd	median	trimmed	mad	min	max	range	skew	kurtosis	se	GrubbsP
sex	1272	1,00	0,00	1,00	1,00	0,00	1,00	1,00	0,00			0,00	
age	1272	32,92	9,44	31,00	32,09	8,90	18,00	77,00	59,00	0,88	0,89	0,26	
orientation	1272	1,00	0,00	1,00	1,00	0,00	1,00	1,00	0,00			0,00	
freq	1272	-0,04	1,00	-0,08	0,05	1,36	-3,03	1,14	4,18	-0,49	-0,34	0,03	1,00
s_iat_sex1_loss	1272	0,00	1,03	0,15	0,01	1,31	-1,71	2,31	4,01	0,08	-0,53	0,03	1,00
s_iat_sex2_loss	1272	0,07	0,92	-0,66	-0,06	0,00	-0,66	2,82	3,49	0,89	-0,25	0,03	1,00
s_iat_sex3_loss	1272	0,08	0,87	-0,49	-0,08	0,00	-0,49	3,07	3,57	1,20	0,31	0,02	0,33
s_iat_sex4_cra	1272	0,07	0,92	-0,67	-0,07	0,00	-0,67	2,40	3,07	0,86	-0,45	0,03	1,00
s_iat_sex5_cra	1272	0,08	0,80	-0,33	-0,09	0,00	-0,33	2,87	3,20	1,66	1,48	0,02	0,30
s_iat_sex6_loss	1272	0,05	0,97	0,20	-0,06	1,65	-0,92	2,63	3,55	0,59	-0,57	0,03	1,00
s_iat_sex7_cra	1272	0,05	0,96	0,30	-0,05	1,36	-0,89	3,07	3,97	0,62	-0,47	0,03	0,97
s_iat_sex8_loss	1272	0,05	0,97	0,08	-0,05	1,53	-0,96	2,48	3,43	0,53	-0,69	0,03	1,00
s_iat_sex9_loss	1272	0,08	0,89	-0,55	-0,07	0,00	-0,55	3,07	3,63	1,07	0,02	0,02	0,43
s_iat_sex10_cra	1272	0,08	0,88	-0,54	-0,08	0,00	-0,54	2,32	2,86	1,06	-0,20	0,02	1,00
s_iat_sex11_cra	1272	0,07	0,90	-0,61	-0,06	0,00	-0,61	2,74	3,35	0,97	-0,17	0,03	1,00
s_iat_sex12_cra	1272	0,08	0,81	-0,35	-0,09	0,00	-0,35	3,03	3,39	1,59	1,28	0,02	0,16
with_1	1272	0,07	0,89	-0,58	-0,06	0,00	-0,58	3,07	3,66	1,02	-0,05	0,03	0,57
with_2	1272	0,08	0,79	-0,33	-0,10	0,00	-0,33	3,61	3,93	1,66	1,44	0,02	0,00
with_3	1272	0,08	0,88	-0,53	-0,07	0,00	-0,53	3,07	3,60	1,12	0,12	0,02	0,38
obj_1	1272	-0,03	0,99	-0,03	0,04	1,58	-2,96	1,23	4,19	-0,38	-0,38	0,03	1,00
obj_2	1272	0,01	1,03	0,20	-0,01	1,29	-1,65	2,08	3,73	0,10	-0,63	0,03	1,00
obj_3	1272	-0,01	1,03	-0,34	-0,02	1,44	-2,32	1,78	4,11	-0,11	-0,42	0,03	1,00
obj_4	1272	0,01	1,03	0,36	0,00	1,44	-1,69	2,26	3,95	0,13	-0,50	0,03	1,00
obj_5	1272	0,00	1,03	0,12	-0,02	1,24	-1,66	1,87	3,54	0,06	-0,73	0,03	1,00
