# Supplementary material for: Men’s internet sex addiction predicts sexual objectification of women even after taking pornography consumption frequency into account
Source: Front Psychol. 2025 Feb 12;16:1517317. doi: 10.3389/fpsyg.2025.1517317 (PMC11861099; doi:10.3389/fpsyg.2025.1517317)
Supplement: Supplementary file 3 [file Data_Sheet_3.pdf]

lavaan 0.6-12 ended normally after 169 iterations

Estimator	DWLS
Optimization method	NLMINB
Number of model parameters	94
Number of observations	1272

Model Test User Model:

	Standard	Robust
Test Statistic	1122.754	1200.019
Degrees of freedom	133	133
P-value (Chi-square)	0.000	0.000
Scaling correction factor		0.976
Shift parameter		49.263
simple second-order correction		

Parameter Estimates:

Standard errors	Robust.sem
Information	Expected
Information saturated (h1) model	Unstructured

Latent Variables:

	Estimate	Std.Err	z-value	P(>|z|)	Std.lv	Std.all
loss =~						
s_iat_sex1_lss	0.096	0.131	0.738	0.461	0.691	0.691
s_iat_sex2_lss	0.113	0.154	0.738	0.461	0.811	0.811
s_iat_sex3_lss	0.100	0.135	0.737	0.461	0.714	0.714
s_iat_sex6_lss	0.104	0.141	0.738	0.461	0.743	0.743
s_iat_sex8_lss	0.112	0.152	0.737	0.461	0.804	0.804

```
  s_iat_sex9_lss    0.106   0.143   0.738   0.461   0.756   0.756
 control =~
  s_iat_sex4_cra    0.205   0.041   4.947   0.000   0.583   0.583
  s_iat_sex5_cra    0.235   0.048   4.927   0.000   0.667   0.667
  s_iat_sex7_cra    0.260   0.053   4.876   0.000   0.740   0.740
  s_iat_sex10_cr    0.249   0.050   5.000   0.000   0.709   0.709
  s_iat_sex11_cr    0.269   0.054   4.978   0.000   0.766   0.766
  s_iat_sex12_cr    0.276   0.056   4.939   0.000   0.785   0.785
 objectification =~
  obj_1             0.618   0.021  30.001   0.000   0.678   0.678
  obj_2             0.690   0.017  41.713   0.000   0.757   0.757
  obj_3             0.697   0.016  42.580   0.000   0.764   0.764
  obj_4             0.750   0.016  47.992   0.000   0.823   0.823
  obj_5             0.763   0.015  49.417   0.000   0.836   0.836
 gAddiction =~
  loss              7.091   9.798   0.724   0.469   0.990   0.990
  control           2.663   0.610   4.368   0.000   0.936   0.936

Regressions:
                   Estimate  Std.Err  z-value  P(>|z|)   Std.lv  Std.all
 objectification ~
  gAddiction        0.450    0.037   12.090    0.000    0.411   0.411
  freq              0.000                               0.000   0.000
 freq ~
  gAddiction        0.000                               0.000   0.000

Intercepts:
                   Estimate  Std.Err  z-value  P(>|z|)   Std.lv  Std.all
 .s_iat_sex1_lss    0.000                               0.000   0.000
 .s_iat_sex2_lss    0.000                               0.000   0.000
 .s_iat_sex3_lss    0.000                               0.000   0.000
```

.s_iat_sex6_lss 0.000 0.000 0.000

.s_iat_sex8_lss 0.000 0.000 0.000

.s_iat_sex9_lss 0.000 0.000 0.000

.s_iat_sex4_cra 0.000 0.000 0.000

.s_iat_sex5_cra 0.000 0.000 0.000

.s_iat_sex7_cra 0.000 0.000 0.000

.s_iat_sex10_cr 0.000 0.000 0.000

.s_iat_sex11_cr 0.000 0.000 0.000

.s_iat_sex12_cr 0.000 0.000 0.000

.obj_1 0.000 0.000 0.000

.obj_2 0.000 0.000 0.000

.obj_3 0.000 0.000 0.000

.obj_4 0.000 0.000 0.000

.obj_5 0.000 0.000 0.000

.freq 0.000 0.000 0.000

.loss 0.000 0.000 0.000

.control 0.000 0.000 0.000

.objectificatin 0.000 0.000 0.000

gAddiction 0.000 0.000 0.000

Thresholds:

Estimate Std.Err z-value P(>|z|) Std.lv Std.all

s_t_sx1_lss|t1 -1.106 0.044 -25.017 0.000 -1.106 -1.106

s_t_sx1_lss|t2 -0.288 0.036 -8.061 0.000 -0.288 -0.288

s_t_sx1_lss|t3 0.581 0.037 15.533 0.000 0.581 0.581

s_t_sx1_lss|t4 1.723 0.063 27.551 0.000 1.723 1.723

s_t_sx2_lss|t1 0.150 0.035 4.259 0.000 0.150 0.150

s_t_sx2_lss|t2 0.900 0.041 22.037 0.000 0.900 0.900

s_t_sx2_lss|t3 1.619 0.058 27.785 0.000 1.619 1.619

s_t_sx2_lss|t4 2.239 0.096 23.302 0.000 2.239 2.239

s_t_sx3_lss|t1 0.426 0.036 11.733 0.000 0.426 0.426

s_t_sx3_lss|t2	1.154	0.045	25.568	0.000	1.154	1.154
s_t_sx3_lss|t3	1.860	0.069	26.880	0.000	1.860	1.860
s_t_sx3_lss|t4	2.495	0.125	19.950	0.000	2.495	2.495
s_t_sx6_lss|t1	-0.206	0.035	-5.826	0.000	-0.206	-0.206
s_t_sx6_lss|t2	0.569	0.037	15.259	0.000	0.569	0.569
s_t_sx6_lss|t3	1.343	0.049	27.137	0.000	1.343	1.343
s_t_sx6_lss|t4	2.045	0.080	25.408	0.000	2.045	2.045
s_t_sx8_lss|t1	-0.249	0.036	-7.000	0.000	-0.249	-0.249
s_t_sx8_lss|t2	0.390	0.036	10.790	0.000	0.390	0.390
s_t_sx8_lss|t3	1.067	0.043	24.530	0.000	1.067	1.067
s_t_sx8_lss|t4	1.894	0.071	26.653	0.000	1.894	1.894
s_t_sx9_lss|t1	0.329	0.036	9.176	0.000	0.329	0.329
s_t_sx9_lss|t2	0.980	0.042	23.316	0.000	0.980	0.980
s_t_sx9_lss|t3	1.673	0.060	27.694	0.000	1.673	1.673
s_t_sx9_lss|t4	2.495	0.125	19.950	0.000	2.495	2.495
s_it_sx4_cr|t1	0.142	0.035	4.035	0.000	0.142	0.142
s_it_sx4_cr|t2	0.725	0.039	18.717	0.000	0.725	0.725
s_it_sx4_cr|t3	1.287	0.048	26.767	0.000	1.287	1.287
s_it_sx4_cr|t4	1.817	0.067	27.131	0.000	1.817	1.817
s_it_sx5_cr|t1	0.745	0.039	19.142	0.000	0.745	0.745
s_it_sx5_cr|t2	1.368	0.050	27.274	0.000	1.368	1.368
s_it_sx5_cr|t3	1.787	0.066	27.285	0.000	1.787	1.787
s_it_sx5_cr|t4	2.290	0.101	22.675	0.000	2.290	2.290
s_it_sx7_cr|t1	-0.168	0.035	-4.763	0.000	-0.168	-0.168
s_it_sx7_cr|t2	0.756	0.039	19.353	0.000	0.756	0.756
s_it_sx7_cr|t3	1.550	0.056	27.801	0.000	1.550	1.550
s_it_sx7_cr|t4	2.495	0.125	19.950	0.000	2.495	2.495
s_t_sx10_cr|t1	0.350	0.036	9.733	0.000	0.350	0.350
s_t_sx10_cr|t2	0.886	0.041	21.785	0.000	0.886	0.886
s_t_sx10_cr|t3	1.243	0.047	26.422	0.000	1.243	1.243
s_t_sx10_cr|t4	1.741	0.063	27.487	0.000	1.741	1.741

s_t_sx11_cr|t1	0.231	0.035	6.497	0.000	0.231	0.231
s_t_sx11_cr|t2	0.889	0.041	21.836	0.000	0.889	0.889
s_t_sx11_cr|t3	1.665	0.060	27.712	0.000	1.665	1.665
s_t_sx11_cr|t4	2.151	0.088	24.312	0.000	2.151	2.151
s_t_sx12_cr|t1	0.697	0.038	18.130	0.000	0.697	0.697
s_t_sx12_cr|t2	1.333	0.049	27.079	0.000	1.333	1.333
s_t_sx12_cr|t3	1.894	0.071	26.653	0.000	1.894	1.894
s_t_sx12_cr|t4	2.453	0.120	20.529	0.000	2.453	2.453
obj_1|t1	-2.318	0.104	-22.320	0.000	-2.318	-2.318
obj_1|t2	-1.459	0.053	-27.642	0.000	-1.459	-1.459
obj_1|t3	-0.611	0.038	-16.241	0.000	-0.611	-0.611
obj_1|t4	0.560	0.037	15.040	0.000	0.560	0.560
obj_2|t1	-1.029	0.043	-24.025	0.000	-1.029	-1.029
obj_2|t2	-0.231	0.035	-6.497	0.000	-0.231	-0.231
obj_2|t3	0.633	0.038	16.729	0.000	0.633	0.633
obj_2|t4	1.476	0.053	27.688	0.000	1.476	1.476
obj_3|t1	-1.723	0.063	-27.551	0.000	-1.723	-1.723
obj_3|t2	-0.782	0.039	-19.879	0.000	-0.782	-0.782
obj_3|t3	0.154	0.035	4.371	0.000	0.154	0.154
obj_3|t4	1.178	0.046	25.812	0.000	1.178	1.178
obj_4|t1	-1.070	0.044	-24.575	0.000	-1.070	-1.070
obj_4|t2	-0.119	0.035	-3.363	0.001	-0.119	-0.119
obj_4|t3	0.807	0.040	20.349	0.000	0.807	0.807
obj_4|t4	1.706	0.062	27.606	0.000	1.706	1.706
obj_5|t1	-1.046	0.043	-24.256	0.000	-1.046	-1.046
obj_5|t2	-0.290	0.036	-8.117	0.000	-0.290	-0.290
obj_5|t3	0.533	0.037	14.383	0.000	0.533	0.533
obj_5|t4	1.291	0.048	26.800	0.000	1.291	1.291
freq|t1	-2.453	0.120	-20.529	0.000	-2.453	-2.453
freq|t2	-1.860	0.069	-26.880	0.000	-1.860	-1.860
freq|t3	-1.570	0.056	-27.808	0.000	-1.570	-1.570

	Estimate	Std.Err	z-value	P(>|z|)	Std.lv	Std.all
freq|t4	-1.158	0.045	-25.610	0.000	-1.158	-1.158
freq|t5	-0.650	0.038	-17.108	0.000	-0.650	-0.650
freq|t6	0.479	0.037	13.061	0.000	0.479	0.479

Variances:

	Estimate	Std.Err	z-value	P(>|z|)	Std.lv	Std.all
.s_iat_sex1_lss	0.523				0.523	0.523
.s_iat_sex2_lss	0.342				0.342	0.342
.s_iat_sex3_lss	0.490				0.490	0.490
.s_iat_sex6_lss	0.448				0.448	0.448
.s_iat_sex8_lss	0.354				0.354	0.354
.s_iat_sex9_lss	0.428				0.428	0.428
.s_iat_sex4_cra	0.660				0.660	0.660
.s_iat_sex5_cra	0.555				0.555	0.555
.s_iat_sex7_cra	0.452				0.452	0.452
.s_iat_sex10_cr	0.498				0.498	0.498
.s_iat_sex11_cr	0.413				0.413	0.413
.s_iat_sex12_cr	0.383				0.383	0.383
.obj_1	0.541				0.541	0.541
.obj_2	0.427				0.427	0.427
.obj_3	0.416				0.416	0.416
.obj_4	0.322				0.322	0.322
.obj_5	0.300				0.300	0.300
.freq	1.000				1.000	1.000
.loss	1.000				0.020	0.020
.control	1.000				0.124	0.124
.objectificatin	1.000				0.831	0.831
gAddiction	1.000				1.000	1.000

Scales y*:

	Estimate	Std.Err	z-value	P(>|z|)	Std.lv	Std.all

s_iat_sex1_lss	1.000	1.000	1.000
s_iat_sex2_lss	1.000	1.000	1.000
s_iat_sex3_lss	1.000	1.000	1.000
s_iat_sex6_lss	1.000	1.000	1.000
s_iat_sex8_lss	1.000	1.000	1.000
s_iat_sex9_lss	1.000	1.000	1.000
s_iat_sex4_cra	1.000	1.000	1.000
s_iat_sex5_cra	1.000	1.000	1.000
s_iat_sex7_cra	1.000	1.000	1.000
s_iat_sex10_cr	1.000	1.000	1.000
s_iat_sex11_cr	1.000	1.000	1.000
s_iat_sex12_cr	1.000	1.000	1.000
obj_1	1.000	1.000	1.000
obj_2	1.000	1.000	1.000
obj_3	1.000	1.000	1.000
obj_4	1.000	1.000	1.000
obj_5	1.000	1.000	1.000
freq	1.000	1.000	1.000
