# Supplementary material for: Men’s internet sex addiction predicts sexual objectification of women even after taking pornography consumption frequency into account
Source: Front Psychol. 2025 Feb 12;16:1517317. doi: 10.3389/fpsyg.2025.1517317 (PMC11861099; doi:10.3389/fpsyg.2025.1517317)
Supplement: Supplementary file 4 [file Data_Sheet_4.pdf]

lavaan 0.6-12 ended normally after 24 iterations

Estimator	DWLS
Optimization method	NLMINB
Number of model parameters	92
Number of observations	1272

Model Test User Model:

	Standard	Robust
Test Statistic	3153.821	3075.637
Degrees of freedom	135	135
P-value (Chi-square)	0.000	0.000
Scaling correction factor		1.042
Shift parameter		50.055
simple second-order correction		

Parameter Estimates:

Standard errors	Robust.sem
Information	Expected
Information saturated (h1) model	Unstructured

Latent Variables:

	Estimate	Std.Err	z-value	P(>|z|)	Std.lv	Std.all
loss =~						
s_iat_sex1_lss	0.519	0.012	41.613	0.000	0.734	0.734
s_iat_sex2_lss	0.614	0.011	53.532	0.000	0.868	0.868
s_iat_sex3_lss	0.536	0.016	33.720	0.000	0.758	0.758
s_iat_sex6_lss	0.561	0.013	44.129	0.000	0.793	0.793
s_iat_sex8_lss	0.610	0.010	60.667	0.000	0.863	0.863

s_iat_sex9_lss 0.573 0.014 41.581 0.000 0.810 0.810

control =~

s_iat_sex4_cra 0.454 0.018 24.569 0.000 0.642 0.642

s_iat_sex5_cra 0.518 0.020 25.981 0.000 0.733 0.733

s_iat_sex7_cra 0.591 0.015 40.671 0.000 0.836 0.836

s_iat_sex10_cr 0.559 0.017 32.939 0.000 0.791 0.791

s_iat_sex11_cr 0.606 0.015 41.516 0.000 0.858 0.858

s_iat_sex12_cr 0.607 0.015 40.548 0.000 0.858 0.858

objectification =~

obj_1 0.580 0.022 25.822 0.000 0.677 0.677

obj_2 0.648 0.020 32.130 0.000 0.757 0.757

obj_3 0.655 0.020 32.595 0.000 0.764 0.764

obj_4 0.705 0.020 34.467 0.000 0.823 0.823

obj_5 0.716 0.020 35.033 0.000 0.837 0.837

gAddiction =~

loss 1.000 0.707 0.707

control 1.000 0.707 0.707

Regressions:

Estimate Std.Err z-value P(>|z|) Std.lv Std.all

objectification ~

gAddiction 0.603 0.056 10.728 0.000 0.516 0.516

freq 0.000 0.000 0.000

freq ~

gAddiction 0.000 0.000 0.000

Intercepts:

Estimate Std.Err z-value P(>|z|) Std.lv Std.all

.s_iat_sex1_lss 0.000 0.000 0.000

.s_iat_sex2_lss 0.000 0.000 0.000

.s_iat_sex3_lss 0.000 0.000 0.000

.s_iat_sex6_lss	0.000				0.000	0.000
.s_iat_sex8_lss	0.000				0.000	0.000
.s_iat_sex9_lss	0.000				0.000	0.000
.s_iat_sex4_cra	0.000				0.000	0.000
.s_iat_sex5_cra	0.000				0.000	0.000
.s_iat_sex7_cra	0.000				0.000	0.000
.s_iat_sex10_cr	0.000				0.000	0.000
.s_iat_sex11_cr	0.000				0.000	0.000
.s_iat_sex12_cr	0.000				0.000	0.000
.obj_1	0.000				0.000	0.000
.obj_2	0.000				0.000	0.000
.obj_3	0.000				0.000	0.000
.obj_4	0.000				0.000	0.000
.obj_5	0.000				0.000	0.000
.freq	0.000				0.000	0.000
.loss	0.000				0.000	0.000
.control	0.000				0.000	0.000
.objectificatin	0.000				0.000	0.000
gAddiction	0.000				0.000	0.000

Thresholds:

	Estimate	Std.Err	z-value	P(>|z|)	Std.lv	Std.all
s_t_sx1_lss|t1	-1.106	0.044	-25.017	0.000	-1.106	-1.106
s_t_sx1_lss|t2	-0.288	0.036	-8.061	0.000	-0.288	-0.288
s_t_sx1_lss|t3	0.581	0.037	15.533	0.000	0.581	0.581
s_t_sx1_lss|t4	1.723	0.063	27.551	0.000	1.723	1.723
s_t_sx2_lss|t1	0.150	0.035	4.259	0.000	0.150	0.150
s_t_sx2_lss|t2	0.900	0.041	22.037	0.000	0.900	0.900
s_t_sx2_lss|t3	1.619	0.058	27.785	0.000	1.619	1.619
s_t_sx2_lss|t4	2.239	0.096	23.302	0.000	2.239	2.239
s_t_sx3_lss|t1	0.426	0.036	11.733	0.000	0.426	0.426

s_t_sx3_lss|t2	1.154	0.045	25.568	0.000	1.154	1.154
s_t_sx3_lss|t3	1.860	0.069	26.880	0.000	1.860	1.860
s_t_sx3_lss|t4	2.495	0.125	19.950	0.000	2.495	2.495
s_t_sx6_lss|t1	-0.206	0.035	-5.826	0.000	-0.206	-0.206
s_t_sx6_lss|t2	0.569	0.037	15.259	0.000	0.569	0.569
s_t_sx6_lss|t3	1.343	0.049	27.137	0.000	1.343	1.343
s_t_sx6_lss|t4	2.045	0.080	25.408	0.000	2.045	2.045
s_t_sx8_lss|t1	-0.249	0.036	-7.000	0.000	-0.249	-0.249
s_t_sx8_lss|t2	0.390	0.036	10.790	0.000	0.390	0.390
s_t_sx8_lss|t3	1.067	0.043	24.530	0.000	1.067	1.067
s_t_sx8_lss|t4	1.894	0.071	26.653	0.000	1.894	1.894
s_t_sx9_lss|t1	0.329	0.036	9.176	0.000	0.329	0.329
s_t_sx9_lss|t2	0.980	0.042	23.316	0.000	0.980	0.980
s_t_sx9_lss|t3	1.673	0.060	27.694	0.000	1.673	1.673
s_t_sx9_lss|t4	2.495	0.125	19.950	0.000	2.495	2.495
s_it_sx4_cr|t1	0.142	0.035	4.035	0.000	0.142	0.142
s_it_sx4_cr|t2	0.725	0.039	18.717	0.000	0.725	0.725
s_it_sx4_cr|t3	1.287	0.048	26.767	0.000	1.287	1.287
s_it_sx4_cr|t4	1.817	0.067	27.131	0.000	1.817	1.817
s_it_sx5_cr|t1	0.745	0.039	19.142	0.000	0.745	0.745
s_it_sx5_cr|t2	1.368	0.050	27.274	0.000	1.368	1.368
s_it_sx5_cr|t3	1.787	0.066	27.285	0.000	1.787	1.787
s_it_sx5_cr|t4	2.290	0.101	22.675	0.000	2.290	2.290
s_it_sx7_cr|t1	-0.168	0.035	-4.763	0.000	-0.168	-0.168
s_it_sx7_cr|t2	0.756	0.039	19.353	0.000	0.756	0.756
s_it_sx7_cr|t3	1.550	0.056	27.801	0.000	1.550	1.550
s_it_sx7_cr|t4	2.495	0.125	19.950	0.000	2.495	2.495
s_t_sx10_cr|t1	0.350	0.036	9.733	0.000	0.350	0.350
s_t_sx10_cr|t2	0.886	0.041	21.785	0.000	0.886	0.886
s_t_sx10_cr|t3	1.243	0.047	26.422	0.000	1.243	1.243
s_t_sx10_cr|t4	1.741	0.063	27.487	0.000	1.741	1.741

s_t_sx11_cr|t1	0.231	0.035	6.497	0.000	0.231	0.231
s_t_sx11_cr|t2	0.889	0.041	21.836	0.000	0.889	0.889
s_t_sx11_cr|t3	1.665	0.060	27.712	0.000	1.665	1.665
s_t_sx11_cr|t4	2.151	0.088	24.312	0.000	2.151	2.151
s_t_sx12_cr|t1	0.697	0.038	18.130	0.000	0.697	0.697
s_t_sx12_cr|t2	1.333	0.049	27.079	0.000	1.333	1.333
s_t_sx12_cr|t3	1.894	0.071	26.653	0.000	1.894	1.894
s_t_sx12_cr|t4	2.453	0.120	20.529	0.000	2.453	2.453
obj_1|t1	-2.318	0.104	-22.320	0.000	-2.318	-2.318
obj_1|t2	-1.459	0.053	-27.642	0.000	-1.459	-1.459
obj_1|t3	-0.611	0.038	-16.241	0.000	-0.611	-0.611
obj_1|t4	0.560	0.037	15.040	0.000	0.560	0.560
obj_2|t1	-1.029	0.043	-24.025	0.000	-1.029	-1.029
obj_2|t2	-0.231	0.035	-6.497	0.000	-0.231	-0.231
obj_2|t3	0.633	0.038	16.729	0.000	0.633	0.633
obj_2|t4	1.476	0.053	27.688	0.000	1.476	1.476
obj_3|t1	-1.723	0.063	-27.551	0.000	-1.723	-1.723
obj_3|t2	-0.782	0.039	-19.879	0.000	-0.782	-0.782
obj_3|t3	0.154	0.035	4.371	0.000	0.154	0.154
obj_3|t4	1.178	0.046	25.812	0.000	1.178	1.178
obj_4|t1	-1.070	0.044	-24.575	0.000	-1.070	-1.070
obj_4|t2	-0.119	0.035	-3.363	0.001	-0.119	-0.119
obj_4|t3	0.807	0.040	20.349	0.000	0.807	0.807
obj_4|t4	1.706	0.062	27.606	0.000	1.706	1.706
obj_5|t1	-1.046	0.043	-24.256	0.000	-1.046	-1.046
obj_5|t2	-0.290	0.036	-8.117	0.000	-0.290	-0.290
obj_5|t3	0.533	0.037	14.383	0.000	0.533	0.533
obj_5|t4	1.291	0.048	26.800	0.000	1.291	1.291
freq|t1	-2.453	0.120	-20.529	0.000	-2.453	-2.453
freq|t2	-1.860	0.069	-26.880	0.000	-1.860	-1.860
freq|t3	-1.570	0.056	-27.808	0.000	-1.570	-1.570

	Estimate	Std.Err	z-value	P(>|z|)	Std.lv	Std.all
freq|t4	-1.158	0.045	-25.610	0.000	-1.158	-1.158
freq|t5	-0.650	0.038	-17.108	0.000	-0.650	-0.650
freq|t6	0.479	0.037	13.061	0.000	0.479	0.479

Variances:

	Estimate	Std.Err	z-value	P(>|z|)	Std.lv	Std.all
.s_iat_sex1_lss	0.461				0.461	0.461
.s_iat_sex2_lss	0.246				0.246	0.246
.s_iat_sex3_lss	0.426				0.426	0.426
.s_iat_sex6_lss	0.371				0.371	0.371
.s_iat_sex8_lss	0.256				0.256	0.256
.s_iat_sex9_lss	0.344				0.344	0.344
.s_iat_sex4_cra	0.587				0.587	0.587
.s_iat_sex5_cra	0.463				0.463	0.463
.s_iat_sex7_cra	0.302				0.302	0.302
.s_iat_sex10_cr	0.375				0.375	0.375
.s_iat_sex11_cr	0.265				0.265	0.265
.s_iat_sex12_cr	0.263				0.263	0.263
.obj_1	0.542				0.542	0.542
.obj_2	0.427				0.427	0.427
.obj_3	0.416				0.416	0.416
.obj_4	0.323				0.323	0.323
.obj_5	0.300				0.300	0.300
.freq	1.000				1.000	1.000
.loss	1.000				0.500	0.500
.control	1.000				0.500	0.500
.objectificatin	1.000				0.733	0.733
gAddiction	1.000				1.000	1.000

Scales y*:

	Estimate	Std.Err	z-value	P(>|z|)	Std.lv	Std.all

s_iat_sex1_lss	1.000	1.000	1.000
s_iat_sex2_lss	1.000	1.000	1.000
s_iat_sex3_lss	1.000	1.000	1.000
s_iat_sex6_lss	1.000	1.000	1.000
s_iat_sex8_lss	1.000	1.000	1.000
s_iat_sex9_lss	1.000	1.000	1.000
s_iat_sex4_cra	1.000	1.000	1.000
s_iat_sex5_cra	1.000	1.000	1.000
s_iat_sex7_cra	1.000	1.000	1.000
s_iat_sex10_cr	1.000	1.000	1.000
s_iat_sex11_cr	1.000	1.000	1.000
s_iat_sex12_cr	1.000	1.000	1.000
obj_1	1.000	1.000	1.000
obj_2	1.000	1.000	1.000
obj_3	1.000	1.000	1.000
obj_4	1.000	1.000	1.000
obj_5	1.000	1.000	1.000
freq	1.000	1.000	1.000
