# Supplementary material for: Men’s internet sex addiction predicts sexual objectification of women even after taking pornography consumption frequency into account
Source: Front Psychol. 2025 Feb 12;16:1517317. doi: 10.3389/fpsyg.2025.1517317 (PMC11861099; doi:10.3389/fpsyg.2025.1517317)
Supplement: Supplementary file 5 [file Data_Sheet_5.pdf]

Model 1:

lavaan 0.6-12 ended normally after 22 iterations

Estimator	DWLS
Optimization method	NLMINB
Number of model parameters	94
Number of observations	1272

Model Test User Model:

	Standard	Robust
Test Statistic	558.086	731.675
Degrees of freedom	133	133
P-value (Chi-square)	0.000	0.000
Scaling correction factor		0.806
Shift parameter		39.137
simple second-order correction		

Parameter Estimates:

Standard errors	Robust.sem
Information	Expected
Information saturated (h1) model	Unstructured

Latent Variables:

	Estimate	Std.Err	z-value	P(>|z|)	Std.lv	Std.all
gAddiction =~						
s_iat_sex1_lss	0.686	0.017	39.886	0.000	0.686	0.686
s_iat_sex2_lss	0.801	0.015	54.771	0.000	0.801	0.801
s_iat_sex3_lss	0.705	0.021	34.241	0.000	0.705	0.705
s_iat_sex6_lss	0.735	0.016	45.155	0.000	0.735	0.735

s_iat_sex8_lss	0.792	0.013	60.007	0.000	0.792	0.792
s_iat_sex9_lss	0.745	0.017	43.277	0.000	0.745	0.745
s_iat_sex4_cra	0.566	0.024	23.561	0.000	0.566	0.566
s_iat_sex5_cra	0.650	0.026	25.435	0.000	0.650	0.650
s_iat_sex7_cra	0.720	0.017	42.457	0.000	0.720	0.720
s_iat_sex10_cr	0.687	0.021	33.385	0.000	0.687	0.687
s_iat_sex11_cr	0.744	0.018	41.640	0.000	0.744	0.744
s_iat_sex12_cr	0.766	0.020	38.353	0.000	0.766	0.766
objectification =~						
obj_1	0.613	0.020	30.276	0.000	0.680	0.676
obj_2	0.683	0.016	41.641	0.000	0.757	0.752
obj_3	0.689	0.016	42.943	0.000	0.764	0.759
obj_4	0.739	0.015	48.008	0.000	0.819	0.813
obj_5	0.755	0.015	49.729	0.000	0.837	0.831

Regressions:

	Estimate	Std.Err	z-value	P(>|z|)	Std.lv	Std.all
objectification ~						
gAddiction	0.405	0.037	10.871	0.000	0.365	0.365
freq	0.171	0.035	4.918	0.000	0.154	0.154
freq ~						
gAddiction	0.259	0.031	8.377	0.000	0.259	0.259

Intercepts:

	Estimate	Std.Err	z-value	P(>|z|)	Std.lv	Std.all
.s_iat_sex1_lss	0.000				0.000	0.000
.s_iat_sex2_lss	0.000				0.000	0.000
.s_iat_sex3_lss	0.000				0.000	0.000
.s_iat_sex6_lss	0.000				0.000	0.000
.s_iat_sex8_lss	0.000				0.000	0.000
.s_iat_sex9_lss	0.000				0.000	0.000

.s_iat_sex4_cra	0.000				0.000	0.000
.s_iat_sex5_cra	0.000				0.000	0.000
.s_iat_sex7_cra	0.000				0.000	0.000
.s_iat_sex10_cr	0.000				0.000	0.000
.s_iat_sex11_cr	0.000				0.000	0.000
.s_iat_sex12_cr	0.000				0.000	0.000
.obj_1	0.000				0.000	0.000
.obj_2	0.000				0.000	0.000
.obj_3	0.000				0.000	0.000
.obj_4	0.000				0.000	0.000
.obj_5	0.000				0.000	0.000
.freq	0.000				0.000	0.000
gAddiction	0.000				0.000	0.000
.objectificatin	0.000				0.000	0.000

Thresholds:

	Estimate	Std.Err	z-value	P(>|z|)	Std.lv	Std.all
s_t_sx1_lss|t1	-1.106	0.044	-25.017	0.000	-1.106	-1.106
s_t_sx1_lss|t2	-0.288	0.036	-8.061	0.000	-0.288	-0.288
s_t_sx1_lss|t3	0.581	0.037	15.533	0.000	0.581	0.581
s_t_sx1_lss|t4	1.723	0.063	27.551	0.000	1.723	1.723
s_t_sx2_lss|t1	0.150	0.035	4.259	0.000	0.150	0.150
s_t_sx2_lss|t2	0.900	0.041	22.037	0.000	0.900	0.900
s_t_sx2_lss|t3	1.619	0.058	27.785	0.000	1.619	1.619
s_t_sx2_lss|t4	2.239	0.096	23.302	0.000	2.239	2.239
s_t_sx3_lss|t1	0.426	0.036	11.733	0.000	0.426	0.426
s_t_sx3_lss|t2	1.154	0.045	25.568	0.000	1.154	1.154
s_t_sx3_lss|t3	1.860	0.069	26.880	0.000	1.860	1.860
s_t_sx3_lss|t4	2.495	0.125	19.950	0.000	2.495	2.495
s_t_sx6_lss|t1	-0.206	0.035	-5.826	0.000	-0.206	-0.206
s_t_sx6_lss|t2	0.569	0.037	15.259	0.000	0.569	0.569

s_t_sx6_lss|t3	1.343	0.049	27.137	0.000	1.343	1.343
s_t_sx6_lss|t4	2.045	0.080	25.408	0.000	2.045	2.045
s_t_sx8_lss|t1	-0.249	0.036	-7.000	0.000	-0.249	-0.249
s_t_sx8_lss|t2	0.390	0.036	10.790	0.000	0.390	0.390
s_t_sx8_lss|t3	1.067	0.043	24.530	0.000	1.067	1.067
s_t_sx8_lss|t4	1.894	0.071	26.653	0.000	1.894	1.894
s_t_sx9_lss|t1	0.329	0.036	9.176	0.000	0.329	0.329
s_t_sx9_lss|t2	0.980	0.042	23.316	0.000	0.980	0.980
s_t_sx9_lss|t3	1.673	0.060	27.694	0.000	1.673	1.673
s_t_sx9_lss|t4	2.495	0.125	19.950	0.000	2.495	2.495
s_it_sx4_cr|t1	0.142	0.035	4.035	0.000	0.142	0.142
s_it_sx4_cr|t2	0.725	0.039	18.717	0.000	0.725	0.725
s_it_sx4_cr|t3	1.287	0.048	26.767	0.000	1.287	1.287
s_it_sx4_cr|t4	1.817	0.067	27.131	0.000	1.817	1.817
s_it_sx5_cr|t1	0.745	0.039	19.142	0.000	0.745	0.745
s_it_sx5_cr|t2	1.368	0.050	27.274	0.000	1.368	1.368
s_it_sx5_cr|t3	1.787	0.066	27.285	0.000	1.787	1.787
s_it_sx5_cr|t4	2.290	0.101	22.675	0.000	2.290	2.290
s_it_sx7_cr|t1	-0.168	0.035	-4.763	0.000	-0.168	-0.168
s_it_sx7_cr|t2	0.756	0.039	19.353	0.000	0.756	0.756
s_it_sx7_cr|t3	1.550	0.056	27.801	0.000	1.550	1.550
s_it_sx7_cr|t4	2.495	0.125	19.950	0.000	2.495	2.495
s_t_sx10_cr|t1	0.350	0.036	9.733	0.000	0.350	0.350
s_t_sx10_cr|t2	0.886	0.041	21.785	0.000	0.886	0.886
s_t_sx10_cr|t3	1.243	0.047	26.422	0.000	1.243	1.243
s_t_sx10_cr|t4	1.741	0.063	27.487	0.000	1.741	1.741
s_t_sx11_cr|t1	0.231	0.035	6.497	0.000	0.231	0.231
s_t_sx11_cr|t2	0.889	0.041	21.836	0.000	0.889	0.889
s_t_sx11_cr|t3	1.665	0.060	27.712	0.000	1.665	1.665
s_t_sx11_cr|t4	2.151	0.088	24.312	0.000	2.151	2.151
s_t_sx12_cr|t1	0.697	0.038	18.130	0.000	0.697	0.697

s_t_sx12_cr|t2	1.333	0.049	27.079	0.000	1.333	1.333
s_t_sx12_cr|t3	1.894	0.071	26.653	0.000	1.894	1.894
s_t_sx12_cr|t4	2.453	0.120	20.529	0.000	2.453	2.453
obj_1|t1	-2.318	0.104	-22.320	0.000	-2.318	-2.306
obj_1|t2	-1.459	0.053	-27.642	0.000	-1.459	-1.451
obj_1|t3	-0.611	0.038	-16.241	0.000	-0.611	-0.608
obj_1|t4	0.560	0.037	15.040	0.000	0.560	0.557
obj_2|t1	-1.029	0.043	-24.025	0.000	-1.029	-1.023
obj_2|t2	-0.231	0.035	-6.497	0.000	-0.231	-0.229
obj_2|t3	0.633	0.038	16.729	0.000	0.633	0.629
obj_2|t4	1.476	0.053	27.688	0.000	1.476	1.467
obj_3|t1	-1.723	0.063	-27.551	0.000	-1.723	-1.712
obj_3|t2	-0.782	0.039	-19.879	0.000	-0.782	-0.777
obj_3|t3	0.154	0.035	4.371	0.000	0.154	0.153
obj_3|t4	1.178	0.046	25.812	0.000	1.178	1.170
obj_4|t1	-1.070	0.044	-24.575	0.000	-1.070	-1.062
obj_4|t2	-0.119	0.035	-3.363	0.001	-0.119	-0.118
obj_4|t3	0.807	0.040	20.349	0.000	0.807	0.801
obj_4|t4	1.706	0.062	27.606	0.000	1.706	1.693
obj_5|t1	-1.046	0.043	-24.256	0.000	-1.046	-1.038
obj_5|t2	-0.290	0.036	-8.117	0.000	-0.290	-0.288
obj_5|t3	0.533	0.037	14.383	0.000	0.533	0.528
obj_5|t4	1.291	0.048	26.800	0.000	1.291	1.282
freq|t1	-2.453	0.120	-20.529	0.000	-2.453	-2.453
freq|t2	-1.860	0.069	-26.880	0.000	-1.860	-1.860
freq|t3	-1.570	0.056	-27.808	0.000	-1.570	-1.570
freq|t4	-1.158	0.045	-25.610	0.000	-1.158	-1.158
freq|t5	-0.650	0.038	-17.108	0.000	-0.650	-0.650
freq|t6	0.479	0.037	13.061	0.000	0.479	0.479

Variances:

	Estimate	Std.Err	z-value	P(>|z|)	Std.lv	Std.all
.s_iat_sex1_lss	0.530				0.530	0.530
.s_iat_sex2_lss	0.358				0.358	0.358
.s_iat_sex3_lss	0.503				0.503	0.503
.s_iat_sex6_lss	0.459				0.459	0.459
.s_iat_sex8_lss	0.373				0.373	0.373
.s_iat_sex9_lss	0.445				0.445	0.445
.s_iat_sex4_cra	0.680				0.680	0.680
.s_iat_sex5_cra	0.577				0.577	0.577
.s_iat_sex7_cra	0.482				0.482	0.482
.s_iat_sex10_cr	0.528				0.528	0.528
.s_iat_sex11_cr	0.447				0.447	0.447
.s_iat_sex12_cr	0.413				0.413	0.413
.obj_1	0.548				0.548	0.543
.obj_2	0.439				0.439	0.434
.obj_3	0.430				0.430	0.424
.obj_4	0.344				0.344	0.339
.obj_5	0.315				0.315	0.310
.freq	0.933				0.933	0.933
gAddiction	1.000				1.000	1.000
.objectificatin	1.000				0.814	0.814

Scales y*:

	Estimate	Std.Err	z-value	P(>|z|)	Std.lv	Std.all
s_iat_sex1_lss	1.000				1.000	1.000
s_iat_sex2_lss	1.000				1.000	1.000
s_iat_sex3_lss	1.000				1.000	1.000
s_iat_sex6_lss	1.000				1.000	1.000
s_iat_sex8_lss	1.000				1.000	1.000
s_iat_sex9_lss	1.000				1.000	1.000
s_iat_sex4_cra	1.000				1.000	1.000

```
    s_iat_sex5_cra    1.000                        1.000    1.000
    s_iat_sex7_cra    1.000                        1.000    1.000
    s_iat_sex10_cr    1.000                        1.000    1.000
    s_iat_sex11_cr    1.000                        1.000    1.000
    s_iat_sex12_cr    1.000                        1.000    1.000
    obj_1             1.000                        1.000    1.000
    obj_2             1.000                        1.000    1.000
    obj_3             1.000                        1.000    1.000
    obj_4             1.000                        1.000    1.000
    obj_5             1.000                        1.000    1.000
    freq              1.000                        1.000    1.000

> summary(fit3, standardize = TRUE)
lavaan 0.6-12 ended normally after 24 iterations

  Estimator                                  DWLS
  Optimization method                      NLMINB
  Number of model parameters                   92

  Number of observations                     1272

Model Test User Model:
                                        Standard      Robust
  Test Statistic                        3153.821    3075.637
  Degrees of freedom                         135         135
  P-value (Chi-square)                     0.000       0.000
  Scaling correction factor                            1.042
  Shift parameter                                     50.055
    simple second-order correction

Parameter Estimates:
```

Standard errors	Robust.sem
Information	Expected
Information saturated (h1) model	Unstructured

Latent Variables:

	Estimate	Std.Err	z-value	P(>|z|)	Std.lv	Std.all
loss =~						
s_iat_sex1_lss	0.519	0.012	41.613	0.000	0.734	0.734
s_iat_sex2_lss	0.614	0.011	53.532	0.000	0.868	0.868
s_iat_sex3_lss	0.536	0.016	33.720	0.000	0.758	0.758
s_iat_sex6_lss	0.561	0.013	44.129	0.000	0.793	0.793
s_iat_sex8_lss	0.610	0.010	60.667	0.000	0.863	0.863
s_iat_sex9_lss	0.573	0.014	41.581	0.000	0.810	0.810
control =~						
s_iat_sex4_cra	0.454	0.018	24.569	0.000	0.642	0.642
s_iat_sex5_cra	0.518	0.020	25.981	0.000	0.733	0.733
s_iat_sex7_cra	0.591	0.015	40.671	0.000	0.836	0.836
s_iat_sex10_cr	0.559	0.017	32.939	0.000	0.791	0.791
s_iat_sex11_cr	0.606	0.015	41.516	0.000	0.858	0.858
s_iat_sex12_cr	0.607	0.015	40.548	0.000	0.858	0.858
objectification =~						
obj_1	0.580	0.022	25.822	0.000	0.677	0.677
obj_2	0.648	0.020	32.130	0.000	0.757	0.757
obj_3	0.655	0.020	32.595	0.000	0.764	0.764
obj_4	0.705	0.020	34.467	0.000	0.823	0.823
obj_5	0.716	0.020	35.033	0.000	0.837	0.837
gAddiction =~						
loss	1.000				0.707	0.707
control	1.000				0.707	0.707

Regressions:

	Estimate	Std.Err	z-value	P(>|z|)	Std.lv	Std.all
objectification ~						
gAddiction	0.603	0.056	10.728	0.000	0.516	0.516
freq	0.000				0.000	0.000
freq ~						
gAddiction	0.000				0.000	0.000

Intercepts:

	Estimate	Std.Err	z-value	P(>|z|)	Std.lv	Std.all
.s_iat_sex1_lss	0.000				0.000	0.000
.s_iat_sex2_lss	0.000				0.000	0.000
.s_iat_sex3_lss	0.000				0.000	0.000
.s_iat_sex6_lss	0.000				0.000	0.000
.s_iat_sex8_lss	0.000				0.000	0.000
.s_iat_sex9_lss	0.000				0.000	0.000
.s_iat_sex4_cra	0.000				0.000	0.000
.s_iat_sex5_cra	0.000				0.000	0.000
.s_iat_sex7_cra	0.000				0.000	0.000
.s_iat_sex10_cr	0.000				0.000	0.000
.s_iat_sex11_cr	0.000				0.000	0.000
.s_iat_sex12_cr	0.000				0.000	0.000
.obj_1	0.000				0.000	0.000
.obj_2	0.000				0.000	0.000
.obj_3	0.000				0.000	0.000
.obj_4	0.000				0.000	0.000
.obj_5	0.000				0.000	0.000
.freq	0.000				0.000	0.000
.loss	0.000				0.000	0.000
.control	0.000				0.000	0.000
.objectificatin	0.000				0.000	0.000

gAddiction	0.000				0.000	0.000

Thresholds:

	Estimate	Std.Err	z-value	P(>|z|)	Std.lv	Std.all
s_t_sx1_lss|t1	-1.106	0.044	-25.017	0.000	-1.106	-1.106
s_t_sx1_lss|t2	-0.288	0.036	-8.061	0.000	-0.288	-0.288
s_t_sx1_lss|t3	0.581	0.037	15.533	0.000	0.581	0.581
s_t_sx1_lss|t4	1.723	0.063	27.551	0.000	1.723	1.723
s_t_sx2_lss|t1	0.150	0.035	4.259	0.000	0.150	0.150
s_t_sx2_lss|t2	0.900	0.041	22.037	0.000	0.900	0.900
s_t_sx2_lss|t3	1.619	0.058	27.785	0.000	1.619	1.619
s_t_sx2_lss|t4	2.239	0.096	23.302	0.000	2.239	2.239
s_t_sx3_lss|t1	0.426	0.036	11.733	0.000	0.426	0.426
s_t_sx3_lss|t2	1.154	0.045	25.568	0.000	1.154	1.154
s_t_sx3_lss|t3	1.860	0.069	26.880	0.000	1.860	1.860
s_t_sx3_lss|t4	2.495	0.125	19.950	0.000	2.495	2.495
s_t_sx6_lss|t1	-0.206	0.035	-5.826	0.000	-0.206	-0.206
s_t_sx6_lss|t2	0.569	0.037	15.259	0.000	0.569	0.569
s_t_sx6_lss|t3	1.343	0.049	27.137	0.000	1.343	1.343
s_t_sx6_lss|t4	2.045	0.080	25.408	0.000	2.045	2.045
s_t_sx8_lss|t1	-0.249	0.036	-7.000	0.000	-0.249	-0.249
s_t_sx8_lss|t2	0.390	0.036	10.790	0.000	0.390	0.390
s_t_sx8_lss|t3	1.067	0.043	24.530	0.000	1.067	1.067
s_t_sx8_lss|t4	1.894	0.071	26.653	0.000	1.894	1.894
s_t_sx9_lss|t1	0.329	0.036	9.176	0.000	0.329	0.329
s_t_sx9_lss|t2	0.980	0.042	23.316	0.000	0.980	0.980
s_t_sx9_lss|t3	1.673	0.060	27.694	0.000	1.673	1.673
s_t_sx9_lss|t4	2.495	0.125	19.950	0.000	2.495	2.495
s_it_sx4_cr|t1	0.142	0.035	4.035	0.000	0.142	0.142
s_it_sx4_cr|t2	0.725	0.039	18.717	0.000	0.725	0.725
s_it_sx4_cr|t3	1.287	0.048	26.767	0.000	1.287	1.287

s_it_sx4_cr|t4	1.817	0.067	27.131	0.000	1.817	1.817
s_it_sx5_cr|t1	0.745	0.039	19.142	0.000	0.745	0.745
s_it_sx5_cr|t2	1.368	0.050	27.274	0.000	1.368	1.368
s_it_sx5_cr|t3	1.787	0.066	27.285	0.000	1.787	1.787
s_it_sx5_cr|t4	2.290	0.101	22.675	0.000	2.290	2.290
s_it_sx7_cr|t1	-0.168	0.035	-4.763	0.000	-0.168	-0.168
s_it_sx7_cr|t2	0.756	0.039	19.353	0.000	0.756	0.756
s_it_sx7_cr|t3	1.550	0.056	27.801	0.000	1.550	1.550
s_it_sx7_cr|t4	2.495	0.125	19.950	0.000	2.495	2.495
s_t_sx10_cr|t1	0.350	0.036	9.733	0.000	0.350	0.350
s_t_sx10_cr|t2	0.886	0.041	21.785	0.000	0.886	0.886
s_t_sx10_cr|t3	1.243	0.047	26.422	0.000	1.243	1.243
s_t_sx10_cr|t4	1.741	0.063	27.487	0.000	1.741	1.741
s_t_sx11_cr|t1	0.231	0.035	6.497	0.000	0.231	0.231
s_t_sx11_cr|t2	0.889	0.041	21.836	0.000	0.889	0.889
s_t_sx11_cr|t3	1.665	0.060	27.712	0.000	1.665	1.665
s_t_sx11_cr|t4	2.151	0.088	24.312	0.000	2.151	2.151
s_t_sx12_cr|t1	0.697	0.038	18.130	0.000	0.697	0.697
s_t_sx12_cr|t2	1.333	0.049	27.079	0.000	1.333	1.333
s_t_sx12_cr|t3	1.894	0.071	26.653	0.000	1.894	1.894
s_t_sx12_cr|t4	2.453	0.120	20.529	0.000	2.453	2.453
obj_1|t1	-2.318	0.104	-22.320	0.000	-2.318	-2.318
obj_1|t2	-1.459	0.053	-27.642	0.000	-1.459	-1.459
obj_1|t3	-0.611	0.038	-16.241	0.000	-0.611	-0.611
obj_1|t4	0.560	0.037	15.040	0.000	0.560	0.560
obj_2|t1	-1.029	0.043	-24.025	0.000	-1.029	-1.029
obj_2|t2	-0.231	0.035	-6.497	0.000	-0.231	-0.231
obj_2|t3	0.633	0.038	16.729	0.000	0.633	0.633
obj_2|t4	1.476	0.053	27.688	0.000	1.476	1.476
obj_3|t1	-1.723	0.063	-27.551	0.000	-1.723	-1.723
obj_3|t2	-0.782	0.039	-19.879	0.000	-0.782	-0.782

```
obj_3|t3      0.154   0.035   4.371   0.000   0.154   0.154
obj_3|t4      1.178   0.046  25.812   0.000   1.178   1.178
obj_4|t1     -1.070   0.044 -24.575   0.000  -1.070  -1.070
obj_4|t2     -0.119   0.035  -3.363   0.001  -0.119  -0.119
obj_4|t3      0.807   0.040  20.349   0.000   0.807   0.807
obj_4|t4      1.706   0.062  27.606   0.000   1.706   1.706
obj_5|t1     -1.046   0.043 -24.256   0.000  -1.046  -1.046
obj_5|t2     -0.290   0.036  -8.117   0.000  -0.290  -0.290
obj_5|t3      0.533   0.037  14.383   0.000   0.533   0.533
obj_5|t4      1.291   0.048  26.800   0.000   1.291   1.291
freq|t1      -2.453   0.120 -20.529   0.000  -2.453  -2.453
freq|t2      -1.860   0.069 -26.880   0.000  -1.860  -1.860
freq|t3      -1.570   0.056 -27.808   0.000  -1.570  -1.570
freq|t4      -1.158   0.045 -25.610   0.000  -1.158  -1.158
freq|t5      -0.650   0.038 -17.108   0.000  -0.650  -0.650
freq|t6       0.479   0.037  13.061   0.000   0.479   0.479

Variances:
                   Estimate  Std.Err  z-value  P(>|z|)   Std.lv  Std.all
   .s_iat_sex1_lss    0.461                               0.461    0.461
   .s_iat_sex2_lss    0.246                               0.246    0.246
   .s_iat_sex3_lss    0.426                               0.426    0.426
   .s_iat_sex6_lss    0.371                               0.371    0.371
   .s_iat_sex8_lss    0.256                               0.256    0.256
   .s_iat_sex9_lss    0.344                               0.344    0.344
   .s_iat_sex4_cra    0.587                               0.587    0.587
   .s_iat_sex5_cra    0.463                               0.463    0.463
   .s_iat_sex7_cra    0.302                               0.302    0.302
   .s_iat_sex10_cr    0.375                               0.375    0.375
   .s_iat_sex11_cr    0.265                               0.265    0.265
   .s_iat_sex12_cr    0.263                               0.263    0.263
```

.obj_1	0.542				0.542	0.542
.obj_2	0.427				0.427	0.427
.obj_3	0.416				0.416	0.416
.obj_4	0.323				0.323	0.323
.obj_5	0.300				0.300	0.300
.freq	1.000				1.000	1.000
.loss	1.000				0.500	0.500
.control	1.000				0.500	0.500
.objectificatin	1.000				0.733	0.733
gAddiction	1.000				1.000	1.000

Scales y*:

	Estimate	Std.Err	z-value	P(>|z|)	Std.lv	Std.all
s_iat_sex1_lss	1.000				1.000	1.000
s_iat_sex2_lss	1.000				1.000	1.000
s_iat_sex3_lss	1.000				1.000	1.000
s_iat_sex6_lss	1.000				1.000	1.000
s_iat_sex8_lss	1.000				1.000	1.000
s_iat_sex9_lss	1.000				1.000	1.000
s_iat_sex4_cra	1.000				1.000	1.000
s_iat_sex5_cra	1.000				1.000	1.000
s_iat_sex7_cra	1.000				1.000	1.000
s_iat_sex10_cr	1.000				1.000	1.000
s_iat_sex11_cr	1.000				1.000	1.000
s_iat_sex12_cr	1.000				1.000	1.000
obj_1	1.000				1.000	1.000
obj_2	1.000				1.000	1.000
obj_3	1.000				1.000	1.000
obj_4	1.000				1.000	1.000
obj_5	1.000				1.000	1.000
freq	1.000				1.000	1.000

```
> summary(fit5, standardize = TRUE)
lavaan 0.6-12 ended normally after 21 iterations

  Estimator                                DWLS
  Optimization method                    NLMINB
  Number of model parameters                 92

  Number of observations                   1272

Model Test User Model:
                                   Standard      Robust
  Test Statistic                   1159.320    1215.950
  Degrees of freedom                    135         135
  P-value (Chi-square)                0.000       0.000
  Scaling correction factor                       0.994
  Shift parameter                                50.031
    simple second-order correction

Parameter Estimates:

  Standard errors                        Robust.sem
  Information                              Expected
  Information saturated (h1) model     Unstructured

Latent Variables:
                   Estimate  Std.Err  z-value  P(>|z|)   Std.lv  Std.all
  gAddiction =~
    s_iat_sex1_lss    0.683    0.017   39.668    0.000    0.683    0.683
    s_iat_sex2_lss    0.801    0.015   54.521    0.000    0.801    0.801
    s_iat_sex3_lss    0.706    0.021   34.328    0.000    0.706    0.706
```

s_iat_sex6_lss	0.734	0.016	44.826	0.000	0.734	0.734
s_iat_sex8_lss	0.794	0.013	60.325	0.000	0.794	0.794
s_iat_sex9_lss	0.747	0.017	43.439	0.000	0.747	0.747
s_iat_sex4_cra	0.567	0.024	23.652	0.000	0.567	0.567
s_iat_sex5_cra	0.650	0.026	25.350	0.000	0.650	0.650
s_iat_sex7_cra	0.718	0.017	42.204	0.000	0.718	0.718
s_iat_sex10_cr	0.689	0.021	33.584	0.000	0.689	0.689
s_iat_sex11_cr	0.744	0.018	41.544	0.000	0.744	0.744
s_iat_sex12_cr	0.766	0.020	38.234	0.000	0.766	0.766
objectification =~						
obj_1	0.619	0.020	30.220	0.000	0.677	0.677
obj_2	0.692	0.016	42.208	0.000	0.757	0.757
obj_3	0.699	0.016	43.152	0.000	0.764	0.764
obj_4	0.753	0.015	48.873	0.000	0.823	0.823
obj_5	0.765	0.015	50.452	0.000	0.837	0.837

Regressions:

	Estimate	Std.Err	z-value	P(>|z|)	Std.lv	Std.all
objectification ~						
gAddiction	0.443	0.036	12.251	0.000	0.405	0.405
freq	0.000				0.000	0.000
freq ~						
gAddiction	0.000				0.000	0.000

Intercepts:

	Estimate	Std.Err	z-value	P(>|z|)	Std.lv	Std.all
.s_iat_sex1_lss	0.000				0.000	0.000
.s_iat_sex2_lss	0.000				0.000	0.000
.s_iat_sex3_lss	0.000				0.000	0.000
.s_iat_sex6_lss	0.000				0.000	0.000
.s_iat_sex8_lss	0.000				0.000	0.000

.s_iat_sex9_lss	0.000				0.000	0.000
.s_iat_sex4_cra	0.000				0.000	0.000
.s_iat_sex5_cra	0.000				0.000	0.000
.s_iat_sex7_cra	0.000				0.000	0.000
.s_iat_sex10_cr	0.000				0.000	0.000
.s_iat_sex11_cr	0.000				0.000	0.000
.s_iat_sex12_cr	0.000				0.000	0.000
.obj_1	0.000				0.000	0.000
.obj_2	0.000				0.000	0.000
.obj_3	0.000				0.000	0.000
.obj_4	0.000				0.000	0.000
.obj_5	0.000				0.000	0.000
.freq	0.000				0.000	0.000
gAddiction	0.000				0.000	0.000
.objectificatin	0.000				0.000	0.000

Thresholds:

	Estimate	Std.Err	z-value	P(>|z|)	Std.lv	Std.all
s_t_sx1_lss|t1	-1.106	0.044	-25.017	0.000	-1.106	-1.106
s_t_sx1_lss|t2	-0.288	0.036	-8.061	0.000	-0.288	-0.288
s_t_sx1_lss|t3	0.581	0.037	15.533	0.000	0.581	0.581
s_t_sx1_lss|t4	1.723	0.063	27.551	0.000	1.723	1.723
s_t_sx2_lss|t1	0.150	0.035	4.259	0.000	0.150	0.150
s_t_sx2_lss|t2	0.900	0.041	22.037	0.000	0.900	0.900
s_t_sx2_lss|t3	1.619	0.058	27.785	0.000	1.619	1.619
s_t_sx2_lss|t4	2.239	0.096	23.302	0.000	2.239	2.239
s_t_sx3_lss|t1	0.426	0.036	11.733	0.000	0.426	0.426
s_t_sx3_lss|t2	1.154	0.045	25.568	0.000	1.154	1.154
s_t_sx3_lss|t3	1.860	0.069	26.880	0.000	1.860	1.860
s_t_sx3_lss|t4	2.495	0.125	19.950	0.000	2.495	2.495
s_t_sx6_lss|t1	-0.206	0.035	-5.826	0.000	-0.206	-0.206

s_t_sx6_lss|t2	0.569	0.037	15.259	0.000	0.569	0.569
s_t_sx6_lss|t3	1.343	0.049	27.137	0.000	1.343	1.343
s_t_sx6_lss|t4	2.045	0.080	25.408	0.000	2.045	2.045
s_t_sx8_lss|t1	-0.249	0.036	-7.000	0.000	-0.249	-0.249
s_t_sx8_lss|t2	0.390	0.036	10.790	0.000	0.390	0.390
s_t_sx8_lss|t3	1.067	0.043	24.530	0.000	1.067	1.067
s_t_sx8_lss|t4	1.894	0.071	26.653	0.000	1.894	1.894
s_t_sx9_lss|t1	0.329	0.036	9.176	0.000	0.329	0.329
s_t_sx9_lss|t2	0.980	0.042	23.316	0.000	0.980	0.980
s_t_sx9_lss|t3	1.673	0.060	27.694	0.000	1.673	1.673
s_t_sx9_lss|t4	2.495	0.125	19.950	0.000	2.495	2.495
s_it_sx4_cr|t1	0.142	0.035	4.035	0.000	0.142	0.142
s_it_sx4_cr|t2	0.725	0.039	18.717	0.000	0.725	0.725
s_it_sx4_cr|t3	1.287	0.048	26.767	0.000	1.287	1.287
s_it_sx4_cr|t4	1.817	0.067	27.131	0.000	1.817	1.817
s_it_sx5_cr|t1	0.745	0.039	19.142	0.000	0.745	0.745
s_it_sx5_cr|t2	1.368	0.050	27.274	0.000	1.368	1.368
s_it_sx5_cr|t3	1.787	0.066	27.285	0.000	1.787	1.787
s_it_sx5_cr|t4	2.290	0.101	22.675	0.000	2.290	2.290
s_it_sx7_cr|t1	-0.168	0.035	-4.763	0.000	-0.168	-0.168
s_it_sx7_cr|t2	0.756	0.039	19.353	0.000	0.756	0.756
s_it_sx7_cr|t3	1.550	0.056	27.801	0.000	1.550	1.550
s_it_sx7_cr|t4	2.495	0.125	19.950	0.000	2.495	2.495
s_t_sx10_cr|t1	0.350	0.036	9.733	0.000	0.350	0.350
s_t_sx10_cr|t2	0.886	0.041	21.785	0.000	0.886	0.886
s_t_sx10_cr|t3	1.243	0.047	26.422	0.000	1.243	1.243
s_t_sx10_cr|t4	1.741	0.063	27.487	0.000	1.741	1.741
s_t_sx11_cr|t1	0.231	0.035	6.497	0.000	0.231	0.231
s_t_sx11_cr|t2	0.889	0.041	21.836	0.000	0.889	0.889
s_t_sx11_cr|t3	1.665	0.060	27.712	0.000	1.665	1.665
s_t_sx11_cr|t4	2.151	0.088	24.312	0.000	2.151	2.151

s_t_sx12_cr|t1	0.697	0.038	18.130	0.000	0.697	0.697
s_t_sx12_cr|t2	1.333	0.049	27.079	0.000	1.333	1.333
s_t_sx12_cr|t3	1.894	0.071	26.653	0.000	1.894	1.894
s_t_sx12_cr|t4	2.453	0.120	20.529	0.000	2.453	2.453
obj_1|t1	-2.318	0.104	-22.320	0.000	-2.318	-2.318
obj_1|t2	-1.459	0.053	-27.642	0.000	-1.459	-1.459
obj_1|t3	-0.611	0.038	-16.241	0.000	-0.611	-0.611
obj_1|t4	0.560	0.037	15.040	0.000	0.560	0.560
obj_2|t1	-1.029	0.043	-24.025	0.000	-1.029	-1.029
obj_2|t2	-0.231	0.035	-6.497	0.000	-0.231	-0.231
obj_2|t3	0.633	0.038	16.729	0.000	0.633	0.633
obj_2|t4	1.476	0.053	27.688	0.000	1.476	1.476
obj_3|t1	-1.723	0.063	-27.551	0.000	-1.723	-1.723
obj_3|t2	-0.782	0.039	-19.879	0.000	-0.782	-0.782
obj_3|t3	0.154	0.035	4.371	0.000	0.154	0.154
obj_3|t4	1.178	0.046	25.812	0.000	1.178	1.178
obj_4|t1	-1.070	0.044	-24.575	0.000	-1.070	-1.070
obj_4|t2	-0.119	0.035	-3.363	0.001	-0.119	-0.119
obj_4|t3	0.807	0.040	20.349	0.000	0.807	0.807
obj_4|t4	1.706	0.062	27.606	0.000	1.706	1.706
obj_5|t1	-1.046	0.043	-24.256	0.000	-1.046	-1.046
obj_5|t2	-0.290	0.036	-8.117	0.000	-0.290	-0.290
obj_5|t3	0.533	0.037	14.383	0.000	0.533	0.533
obj_5|t4	1.291	0.048	26.800	0.000	1.291	1.291
freq|t1	-2.453	0.120	-20.529	0.000	-2.453	-2.453
freq|t2	-1.860	0.069	-26.880	0.000	-1.860	-1.860
freq|t3	-1.570	0.056	-27.808	0.000	-1.570	-1.570
freq|t4	-1.158	0.045	-25.610	0.000	-1.158	-1.158
freq|t5	-0.650	0.038	-17.108	0.000	-0.650	-0.650
freq|t6	0.479	0.037	13.061	0.000	0.479	0.479

Variances:

	Estimate	Std.Err	z-value	P(>|z|)	Std.lv	Std.all
.s_iat_sex1_lss	0.533				0.533	0.533
.s_iat_sex2_lss	0.358				0.358	0.358
.s_iat_sex3_lss	0.502				0.502	0.502
.s_iat_sex6_lss	0.461				0.461	0.461
.s_iat_sex8_lss	0.370				0.370	0.370
.s_iat_sex9_lss	0.443				0.443	0.443
.s_iat_sex4_cra	0.678				0.678	0.678
.s_iat_sex5_cra	0.578				0.578	0.578
.s_iat_sex7_cra	0.484				0.484	0.484
.s_iat_sex10_cr	0.526				0.526	0.526
.s_iat_sex11_cr	0.447				0.447	0.447
.s_iat_sex12_cr	0.414				0.414	0.414
.obj_1	0.541				0.541	0.541
.obj_2	0.427				0.427	0.427
.obj_3	0.416				0.416	0.416
.obj_4	0.322				0.322	0.322
.obj_5	0.300				0.300	0.300
.freq	1.000				1.000	1.000
gAddiction	1.000				1.000	1.000
.objectificatin	1.000				0.836	0.836

Scales y*:

	Estimate	Std.Err	z-value	P(>|z|)	Std.lv	Std.all
s_iat_sex1_lss	1.000				1.000	1.000
s_iat_sex2_lss	1.000				1.000	1.000
s_iat_sex3_lss	1.000				1.000	1.000
s_iat_sex6_lss	1.000				1.000	1.000
s_iat_sex8_lss	1.000				1.000	1.000
s_iat_sex9_lss	1.000				1.000	1.000

s_iat_sex4_cra	1.000		1.000	1.000
s_iat_sex5_cra	1.000		1.000	1.000
s_iat_sex7_cra	1.000		1.000	1.000
s_iat_sex10_cr	1.000		1.000	1.000
s_iat_sex11_cr	1.000		1.000	1.000
s_iat_sex12_cr	1.000		1.000	1.000
obj_1	1.000		1.000	1.000
obj_2	1.000		1.000	1.000
obj_3	1.000		1.000	1.000
obj_4	1.000		1.000	1.000
obj_5	1.000		1.000	1.000
freq	1.000		1.000	1.000

rmsea	tli	df	npar	chisq	rmsea.ci.lower	rmsea.ci.upper
0.077	0.972	135.000	92.000	1159.320	0.073	0.081

cfi	srmr
0.975	0.077

Model 2:

lavaan 0.6-12 ended normally after 22 iterations

Estimator	DWLS
Optimization method	NLMINB
Number of model parameters	94
Number of observations	1272

Model Test User Model:

	Standard	Robust
Test Statistic	558.086	731.675
Degrees of freedom	133	133
P-value (Chi-square)	0.000	0.000
Scaling correction factor		0.806
Shift parameter		39.137
simple second-order correction		

Parameter Estimates:

Standard errors	Robust.sem
Information	Expected
Information saturated (h1) model	Unstructured

Latent Variables:

	Estimate	Std.Err	z-value	P(>|z|)	Std.lv	Std.all
gAddiction =~						
s_iat_sex1_lss	0.686	0.017	39.886	0.000	0.686	0.686
s_iat_sex2_lss	0.801	0.015	54.771	0.000	0.801	0.801
s_iat_sex3_lss	0.705	0.021	34.241	0.000	0.705	0.705
s_iat_sex6_lss	0.735	0.016	45.155	0.000	0.735	0.735

s_iat_sex8_lss	0.792	0.013	60.007	0.000	0.792	0.792
s_iat_sex9_lss	0.745	0.017	43.277	0.000	0.745	0.745
s_iat_sex4_cra	0.566	0.024	23.561	0.000	0.566	0.566
s_iat_sex5_cra	0.650	0.026	25.435	0.000	0.650	0.650
s_iat_sex7_cra	0.720	0.017	42.457	0.000	0.720	0.720
s_iat_sex10_cr	0.687	0.021	33.385	0.000	0.687	0.687
s_iat_sex11_cr	0.744	0.018	41.640	0.000	0.744	0.744
s_iat_sex12_cr	0.766	0.020	38.353	0.000	0.766	0.766
objectification =~						
obj_1	0.613	0.020	30.276	0.000	0.680	0.676
obj_2	0.683	0.016	41.641	0.000	0.757	0.752
obj_3	0.689	0.016	42.943	0.000	0.764	0.759
obj_4	0.739	0.015	48.008	0.000	0.819	0.813
obj_5	0.755	0.015	49.729	0.000	0.837	0.831

Regressions:

	Estimate	Std.Err	z-value	P(>|z|)	Std.lv	Std.all
objectification ~						
gAddiction	0.405	0.037	10.871	0.000	0.365	0.365
freq	0.171	0.035	4.918	0.000	0.154	0.154
freq ~						
gAddiction	0.259	0.031	8.377	0.000	0.259	0.259

Intercepts:

	Estimate	Std.Err	z-value	P(>|z|)	Std.lv	Std.all
.s_iat_sex1_lss	0.000				0.000	0.000
.s_iat_sex2_lss	0.000				0.000	0.000
.s_iat_sex3_lss	0.000				0.000	0.000
.s_iat_sex6_lss	0.000				0.000	0.000
.s_iat_sex8_lss	0.000				0.000	0.000
.s_iat_sex9_lss	0.000				0.000	0.000

.s_iat_sex4_cra	0.000				0.000	0.000
.s_iat_sex5_cra	0.000				0.000	0.000
.s_iat_sex7_cra	0.000				0.000	0.000
.s_iat_sex10_cr	0.000				0.000	0.000
.s_iat_sex11_cr	0.000				0.000	0.000
.s_iat_sex12_cr	0.000				0.000	0.000
.obj_1	0.000				0.000	0.000
.obj_2	0.000				0.000	0.000
.obj_3	0.000				0.000	0.000
.obj_4	0.000				0.000	0.000
.obj_5	0.000				0.000	0.000
.freq	0.000				0.000	0.000
gAddiction	0.000				0.000	0.000
.objectificatin	0.000				0.000	0.000

Thresholds:

	Estimate	Std.Err	z-value	P(>|z|)	Std.lv	Std.all
s_t_sx1_lss|t1	-1.106	0.044	-25.017	0.000	-1.106	-1.106
s_t_sx1_lss|t2	-0.288	0.036	-8.061	0.000	-0.288	-0.288
s_t_sx1_lss|t3	0.581	0.037	15.533	0.000	0.581	0.581
s_t_sx1_lss|t4	1.723	0.063	27.551	0.000	1.723	1.723
s_t_sx2_lss|t1	0.150	0.035	4.259	0.000	0.150	0.150
s_t_sx2_lss|t2	0.900	0.041	22.037	0.000	0.900	0.900
s_t_sx2_lss|t3	1.619	0.058	27.785	0.000	1.619	1.619
s_t_sx2_lss|t4	2.239	0.096	23.302	0.000	2.239	2.239
s_t_sx3_lss|t1	0.426	0.036	11.733	0.000	0.426	0.426
s_t_sx3_lss|t2	1.154	0.045	25.568	0.000	1.154	1.154
s_t_sx3_lss|t3	1.860	0.069	26.880	0.000	1.860	1.860
s_t_sx3_lss|t4	2.495	0.125	19.950	0.000	2.495	2.495
s_t_sx6_lss|t1	-0.206	0.035	-5.826	0.000	-0.206	-0.206
s_t_sx6_lss|t2	0.569	0.037	15.259	0.000	0.569	0.569

s_t_sx6_lss|t3	1.343	0.049	27.137	0.000	1.343	1.343
s_t_sx6_lss|t4	2.045	0.080	25.408	0.000	2.045	2.045
s_t_sx8_lss|t1	-0.249	0.036	-7.000	0.000	-0.249	-0.249
s_t_sx8_lss|t2	0.390	0.036	10.790	0.000	0.390	0.390
s_t_sx8_lss|t3	1.067	0.043	24.530	0.000	1.067	1.067
s_t_sx8_lss|t4	1.894	0.071	26.653	0.000	1.894	1.894
s_t_sx9_lss|t1	0.329	0.036	9.176	0.000	0.329	0.329
s_t_sx9_lss|t2	0.980	0.042	23.316	0.000	0.980	0.980
s_t_sx9_lss|t3	1.673	0.060	27.694	0.000	1.673	1.673
s_t_sx9_lss|t4	2.495	0.125	19.950	0.000	2.495	2.495
s_it_sx4_cr|t1	0.142	0.035	4.035	0.000	0.142	0.142
s_it_sx4_cr|t2	0.725	0.039	18.717	0.000	0.725	0.725
s_it_sx4_cr|t3	1.287	0.048	26.767	0.000	1.287	1.287
s_it_sx4_cr|t4	1.817	0.067	27.131	0.000	1.817	1.817
s_it_sx5_cr|t1	0.745	0.039	19.142	0.000	0.745	0.745
s_it_sx5_cr|t2	1.368	0.050	27.274	0.000	1.368	1.368
s_it_sx5_cr|t3	1.787	0.066	27.285	0.000	1.787	1.787
s_it_sx5_cr|t4	2.290	0.101	22.675	0.000	2.290	2.290
s_it_sx7_cr|t1	-0.168	0.035	-4.763	0.000	-0.168	-0.168
s_it_sx7_cr|t2	0.756	0.039	19.353	0.000	0.756	0.756
s_it_sx7_cr|t3	1.550	0.056	27.801	0.000	1.550	1.550
s_it_sx7_cr|t4	2.495	0.125	19.950	0.000	2.495	2.495
s_t_sx10_cr|t1	0.350	0.036	9.733	0.000	0.350	0.350
s_t_sx10_cr|t2	0.886	0.041	21.785	0.000	0.886	0.886
s_t_sx10_cr|t3	1.243	0.047	26.422	0.000	1.243	1.243
s_t_sx10_cr|t4	1.741	0.063	27.487	0.000	1.741	1.741
s_t_sx11_cr|t1	0.231	0.035	6.497	0.000	0.231	0.231
s_t_sx11_cr|t2	0.889	0.041	21.836	0.000	0.889	0.889
s_t_sx11_cr|t3	1.665	0.060	27.712	0.000	1.665	1.665
s_t_sx11_cr|t4	2.151	0.088	24.312	0.000	2.151	2.151
s_t_sx12_cr|t1	0.697	0.038	18.130	0.000	0.697	0.697

s_t_sx12_cr|t2	1.333	0.049	27.079	0.000	1.333	1.333
s_t_sx12_cr|t3	1.894	0.071	26.653	0.000	1.894	1.894
s_t_sx12_cr|t4	2.453	0.120	20.529	0.000	2.453	2.453
obj_1|t1	-2.318	0.104	-22.320	0.000	-2.318	-2.306
obj_1|t2	-1.459	0.053	-27.642	0.000	-1.459	-1.451
obj_1|t3	-0.611	0.038	-16.241	0.000	-0.611	-0.608
obj_1|t4	0.560	0.037	15.040	0.000	0.560	0.557
obj_2|t1	-1.029	0.043	-24.025	0.000	-1.029	-1.023
obj_2|t2	-0.231	0.035	-6.497	0.000	-0.231	-0.229
obj_2|t3	0.633	0.038	16.729	0.000	0.633	0.629
obj_2|t4	1.476	0.053	27.688	0.000	1.476	1.467
obj_3|t1	-1.723	0.063	-27.551	0.000	-1.723	-1.712
obj_3|t2	-0.782	0.039	-19.879	0.000	-0.782	-0.777
obj_3|t3	0.154	0.035	4.371	0.000	0.154	0.153
obj_3|t4	1.178	0.046	25.812	0.000	1.178	1.170
obj_4|t1	-1.070	0.044	-24.575	0.000	-1.070	-1.062
obj_4|t2	-0.119	0.035	-3.363	0.001	-0.119	-0.118
obj_4|t3	0.807	0.040	20.349	0.000	0.807	0.801
obj_4|t4	1.706	0.062	27.606	0.000	1.706	1.693
obj_5|t1	-1.046	0.043	-24.256	0.000	-1.046	-1.038
obj_5|t2	-0.290	0.036	-8.117	0.000	-0.290	-0.288
obj_5|t3	0.533	0.037	14.383	0.000	0.533	0.528
obj_5|t4	1.291	0.048	26.800	0.000	1.291	1.282
freq|t1	-2.453	0.120	-20.529	0.000	-2.453	-2.453
freq|t2	-1.860	0.069	-26.880	0.000	-1.860	-1.860
freq|t3	-1.570	0.056	-27.808	0.000	-1.570	-1.570
freq|t4	-1.158	0.045	-25.610	0.000	-1.158	-1.158
freq|t5	-0.650	0.038	-17.108	0.000	-0.650	-0.650
freq|t6	0.479	0.037	13.061	0.000	0.479	0.479

Variances:

	Estimate	Std.Err	z-value	P(>|z|)	Std.lv	Std.all
.s_iat_sex1_lss	0.530				0.530	0.530
.s_iat_sex2_lss	0.358				0.358	0.358
.s_iat_sex3_lss	0.503				0.503	0.503
.s_iat_sex6_lss	0.459				0.459	0.459
.s_iat_sex8_lss	0.373				0.373	0.373
.s_iat_sex9_lss	0.445				0.445	0.445
.s_iat_sex4_cra	0.680				0.680	0.680
.s_iat_sex5_cra	0.577				0.577	0.577
.s_iat_sex7_cra	0.482				0.482	0.482
.s_iat_sex10_cr	0.528				0.528	0.528
.s_iat_sex11_cr	0.447				0.447	0.447
.s_iat_sex12_cr	0.413				0.413	0.413
.obj_1	0.548				0.548	0.543
.obj_2	0.439				0.439	0.434
.obj_3	0.430				0.430	0.424
.obj_4	0.344				0.344	0.339
.obj_5	0.315				0.315	0.310
.freq	0.933				0.933	0.933
gAddiction	1.000				1.000	1.000
.objectificatin	1.000				0.814	0.814

Scales y*:

	Estimate	Std.Err	z-value	P(>|z|)	Std.lv	Std.all
s_iat_sex1_lss	1.000				1.000	1.000
s_iat_sex2_lss	1.000				1.000	1.000
s_iat_sex3_lss	1.000				1.000	1.000
s_iat_sex6_lss	1.000				1.000	1.000
s_iat_sex8_lss	1.000				1.000	1.000
s_iat_sex9_lss	1.000				1.000	1.000
s_iat_sex4_cra	1.000				1.000	1.000

s_iat_sex5_cra	1.000		1.000	1.000
s_iat_sex7_cra	1.000		1.000	1.000
s_iat_sex10_cr	1.000		1.000	1.000
s_iat_sex11_cr	1.000		1.000	1.000
s_iat_sex12_cr	1.000		1.000	1.000
obj_1	1.000		1.000	1.000
obj_2	1.000		1.000	1.000
obj_3	1.000		1.000	1.000
obj_4	1.000		1.000	1.000
obj_5	1.000		1.000	1.000
freq	1.000		1.000	1.000

rmsea	tli	df	npar	chisq	rmsea.ci.lower	rmsea.ci.upper
0.050	0.988	133.000	94.000	558.086	0.046	0.054

cfi	srmr
0.990	0.050

Model comparison:

	Df	AIC	BIC	Chisq	Chisq diff	Df diff	Pr(>Chisq)
fit6	133			558.09			
fit5	135			1159.32	128.73	2	< 2.2e-16 ***
